# Supplementary material for: The Evaluation of DHPMs as Biotoxic Agents on Pathogen Bacterial Membranes
Source: Membranes (Basel). 2022 Feb 18;12(2):238. doi: 10.3390/membranes12020238 (PMC8878598; doi:10.3390/membranes12020238)
Supplement: Supplementary file 1 [file membranes-12-00238-s001.zip › membranes-1583160 -new_suppl.pdf]

membranes

Supplementary Materials

The Evaluation of DHPMs as Biotoxic Agents on Pathogen Bacterial Membranes

Barbara Gawdzik [1,*], Paweł Kowalczyk [2,*], Dominik Koszelewski [3], Anna Brodzka [3], Joanna Masternak [1], Karol Kramkowski [4], Aleksandra Wypych [5] and Ryszard Ostaszewski [3]

1 Institute of Chemistry, Jan Kochanowski University, Uniwersytecka 7, 25-406 Kielce, Poland; j.masternak@ujk.edu.pl
2 Department of Animal Nutrition, The Kielanowski Institute of Animal Physiology and Nutrition, Polish Academy of Sciences, Instytucka 3, 05-110 Jabłonna, Poland
3 Institute of Organic Chemistry, Polish Academy of Sciences, Kasprzaka 44/52, 01-224 Warsaw, Poland; dominik.koszelewski@icho.edu.pl (D.K.); anna.brodzka@icho.edu.pl (A.B.); ryszard.ostaszewski@icho.edu.pl (R.O.)
4 Department of Physical Chemistry, Medical University of Bialystok, Kilińskiego 1 Str., 15-089 Białystok, Poland; kkramk@wp.pl
5 Centre for Modern Interdisciplinary Technologies Nicolaus Copernicus University in Torun, ul. Wileńska 4, 87-100 Toruń, Poland; wypych@umk.pl
* Correspondence: b.gawdzik@ujk.edu.pl (B.G.); p.kowalczyk@ifzz.pl (P.K.)

Citation: Gawdzik, B.; Kowalczyk, P.; Koszelewski, D.; Brodzka, A.; Masternak, J.; Kramkowski, K.; Wypych, A.; Ostaszewski, R. The Evaluation of DHPMs as Biotoxic Agents on Pathogen Bacterial Membranes. *Membranes* **2022**, *12*, 238. https://doi.org/10.3390/membranes12020238

Academic Editor: Ionela Andreea Neacsu

Received: 19 January 2022
Accepted: 15 February 2022
Published: 18 February 2022

Publisher's Note: MDPI stays neutral with regard to jurisdictional claims in published maps and institutional affiliations.

Copyright: © 2022 by the authors. Submitted for possible open access publication under the terms and conditions of the Creative Commons Attribution (CC BY) license (https://creativecommons.org/licenses/by/4.0/).

Supplementary Materials

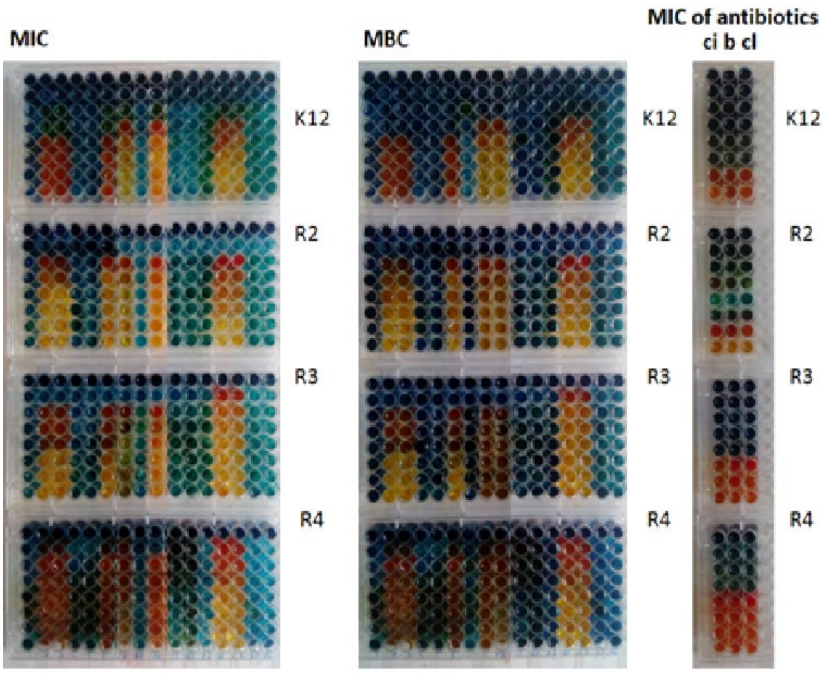

Figure S1. Examples of MIC and MBC on microplates with different concentration of studied compounds (μg/mL^{-1}). Resazurin was added as an indicator of microbial growth with K12, R2, R3, and R4 strains with tested all 16 compounds as described in Figure 2. Additionally, examples of MIC with different strains K12, R2, R3, and R4 of studied antibiotics with ciprofloxacin (ci), bleomycin (b), and cloxacillin (cl) in (μg/mL^{-1}).

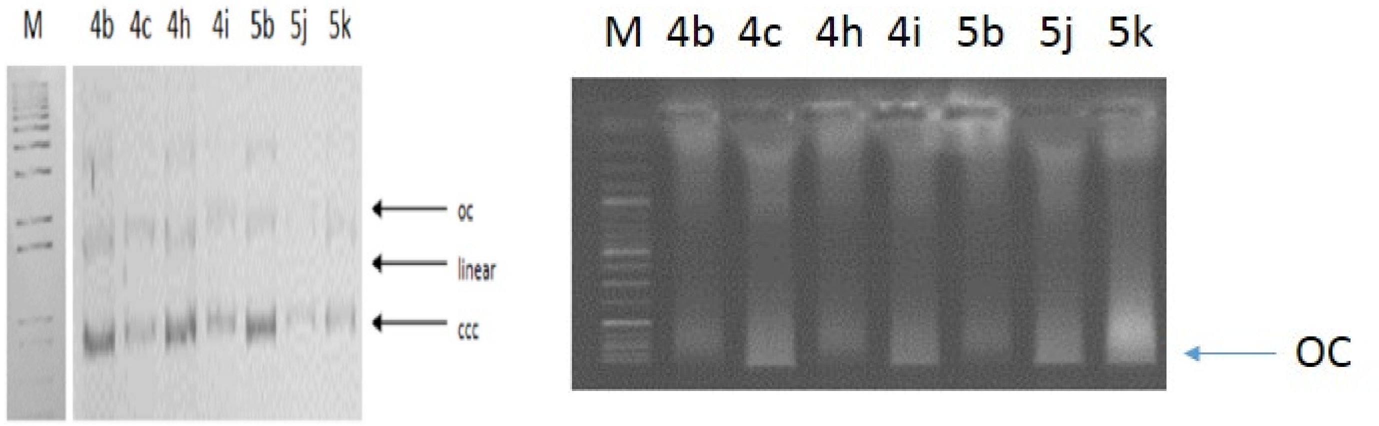

(**a**) (**b**)

Figure S2. An example of an agarose gel electrophoresis separation of isolated plasmids DNA on R4 strains modified with selected coumarin derivatives (Panel A) from 8 selected compounds, as shown in Figure 3, and digested with repair Fpg protein (Panel B). M = marker.

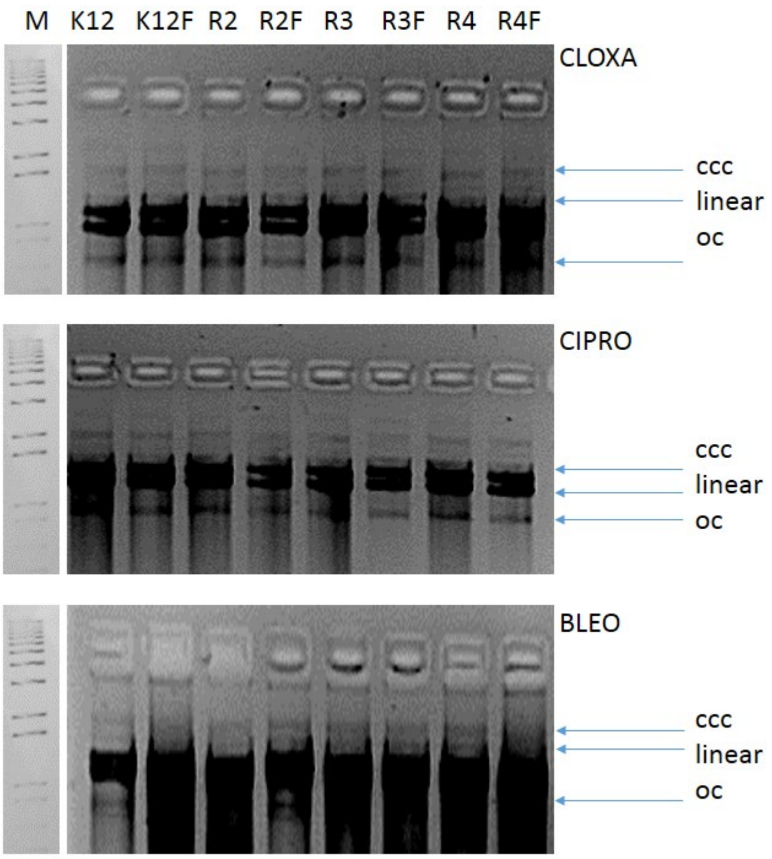

Figure S3. Example of an agarose gel electrophoresis separation of isolated plasmids DNA from K12 and R4 strains modified with antibiotics: bleomycin, ciprofloxacin, and cloxacillin digested (or not) with repair enzymes Fpg. M = marker.

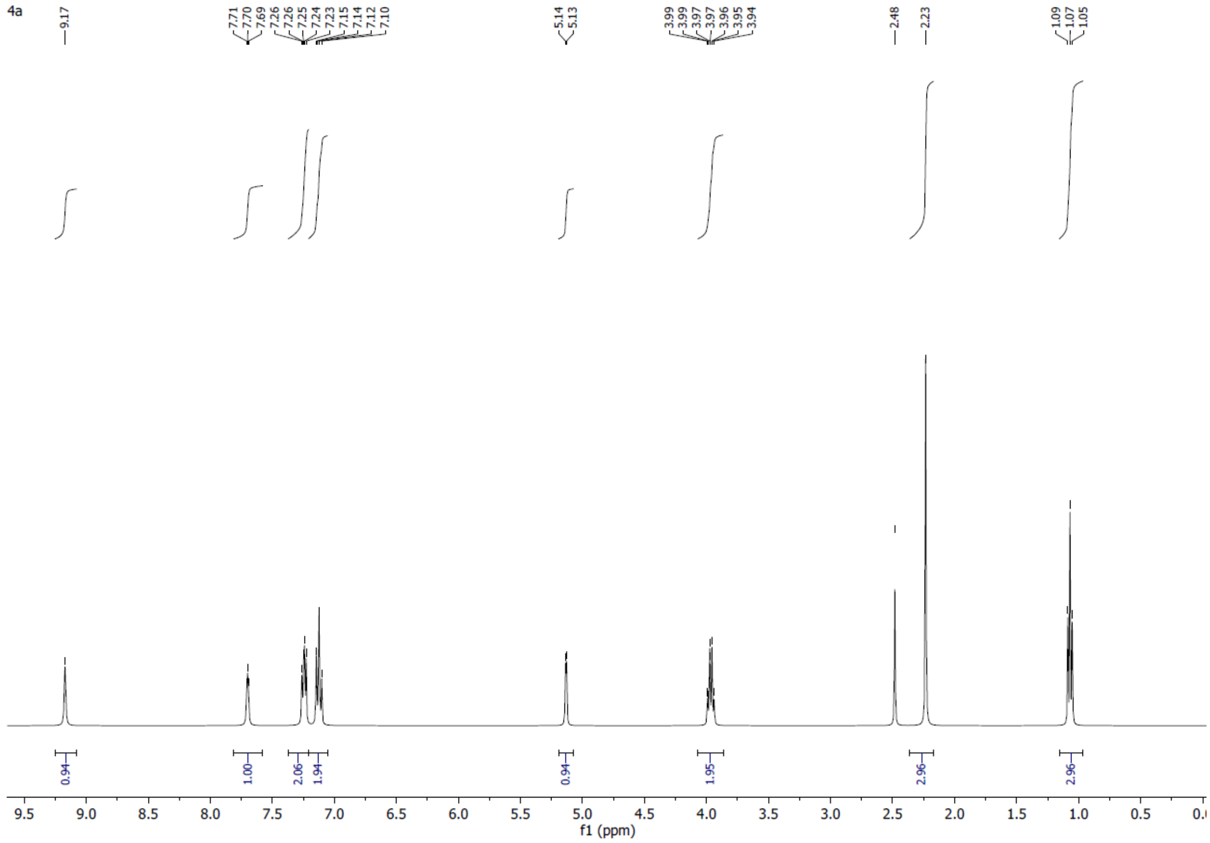

Figure S4. ^{1}H NMR (400 MHz, DMSO-*d*6) of **4a.**

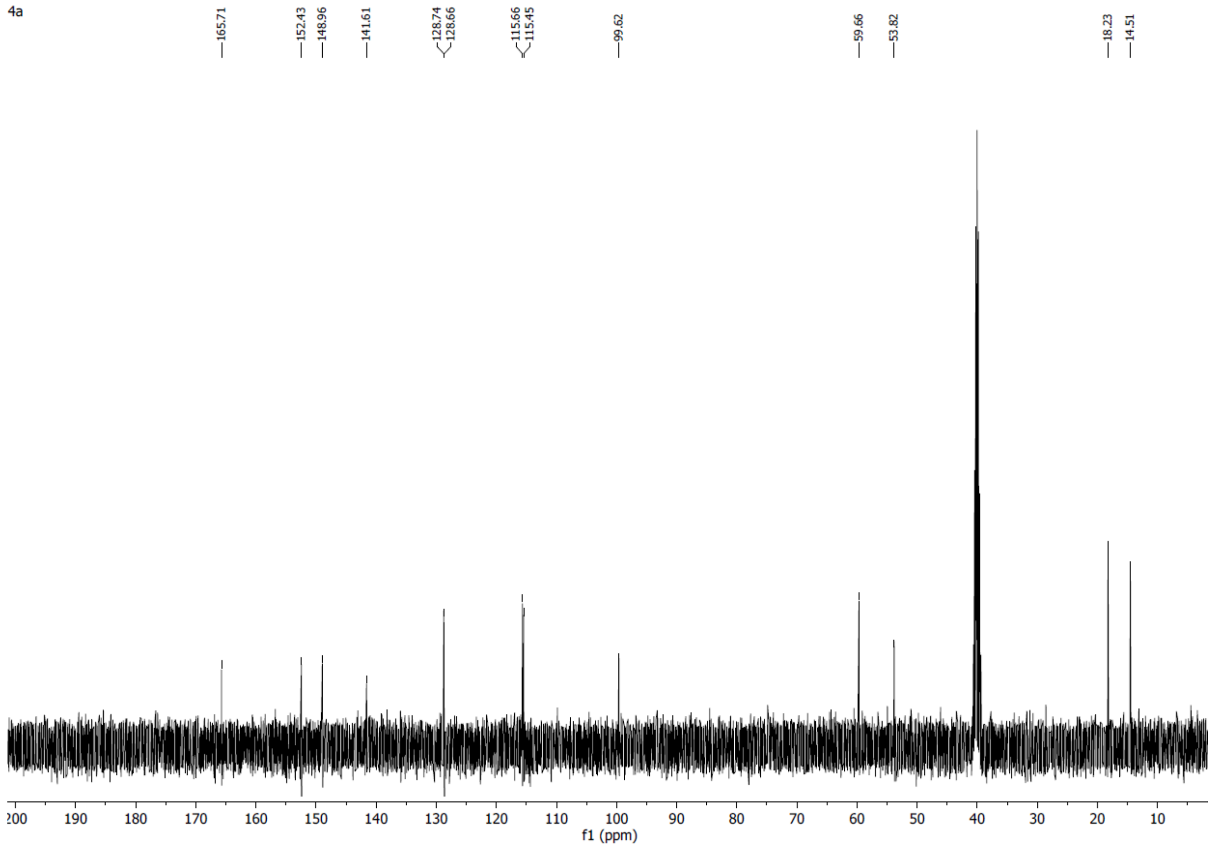

Figure S5. ^{13}C NMR (100 MHz, DMSO-*d*6) of **4a.**

4a

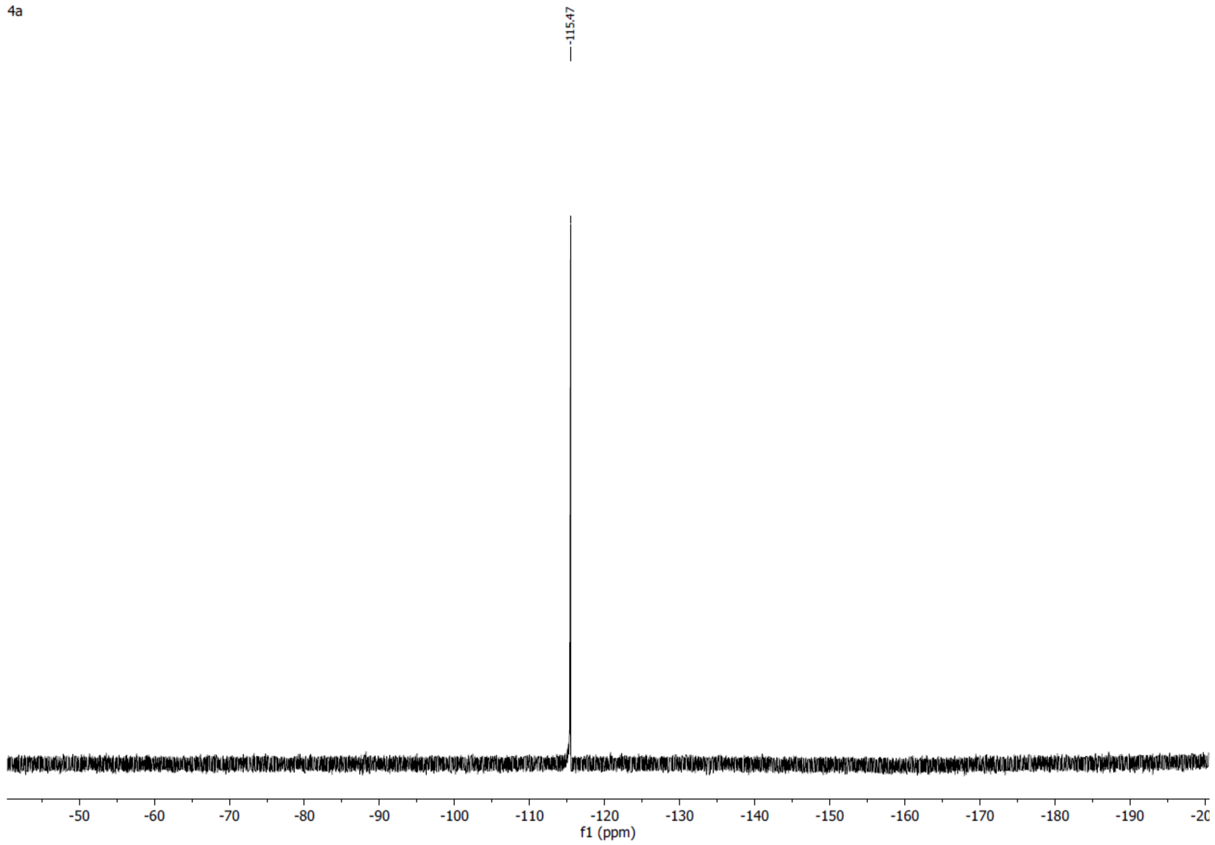

Figure S6. ^{19}F NMR (376 MHz, DMSO-*d*6) of **4a.**

4b

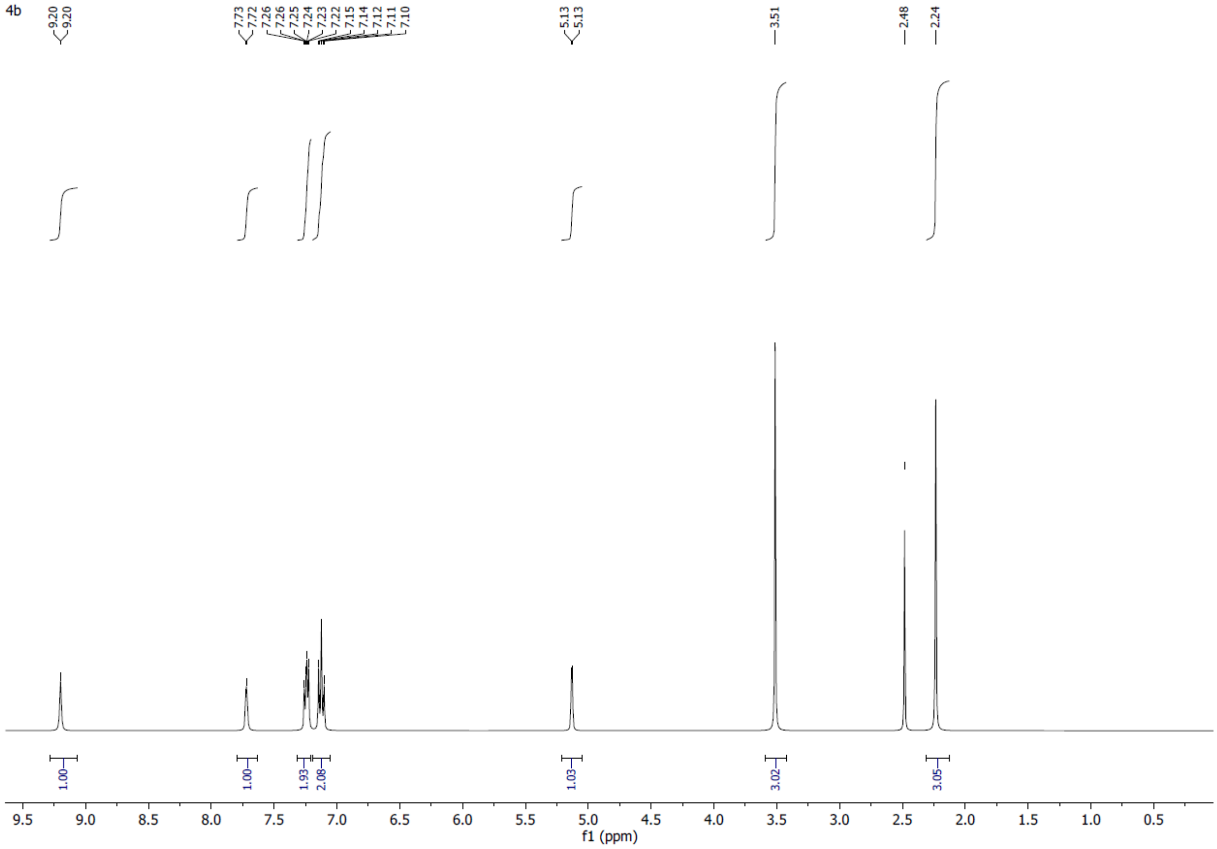

Figure S7. ^{1}H NMR (400 MHz, DMSO-*d*6) of **4b.**

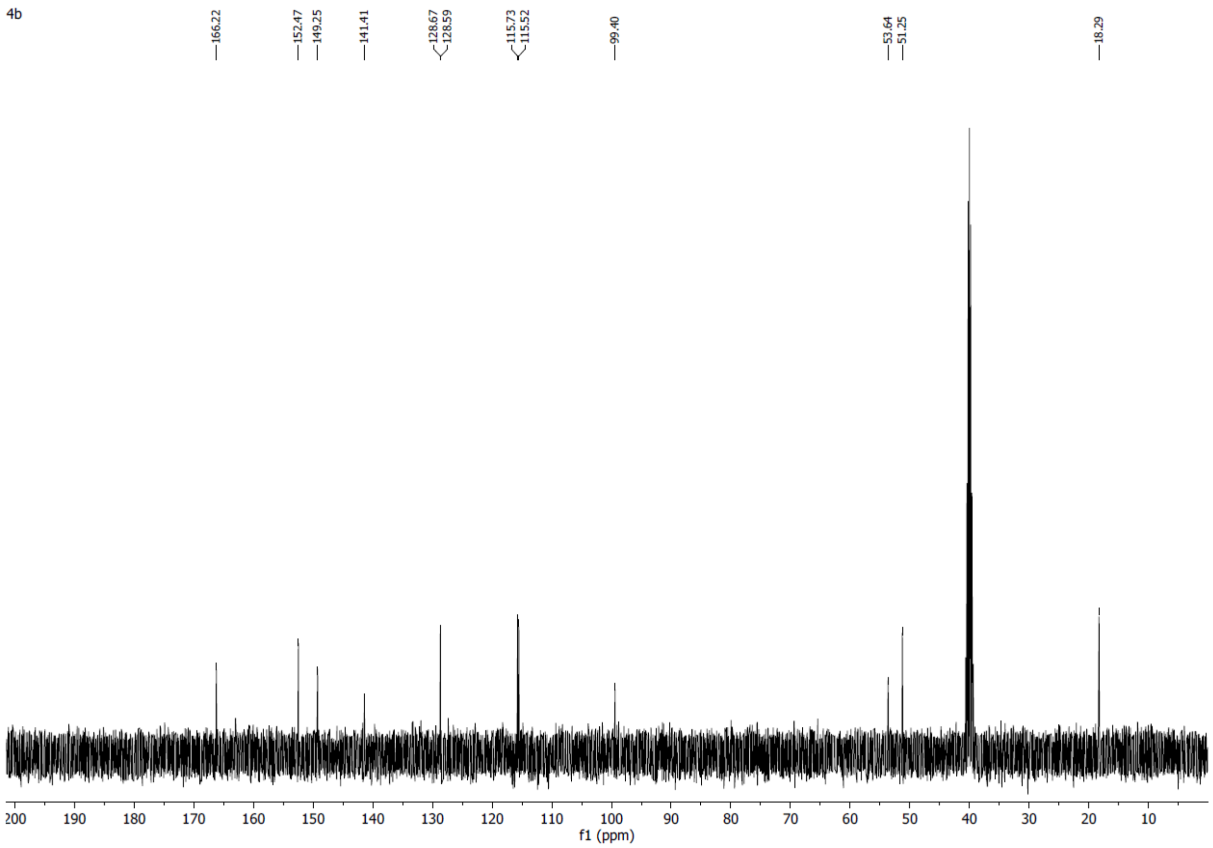

Figure S8. ^{13}C NMR (100 MHz, DMSO-*d*6) of **4b.**

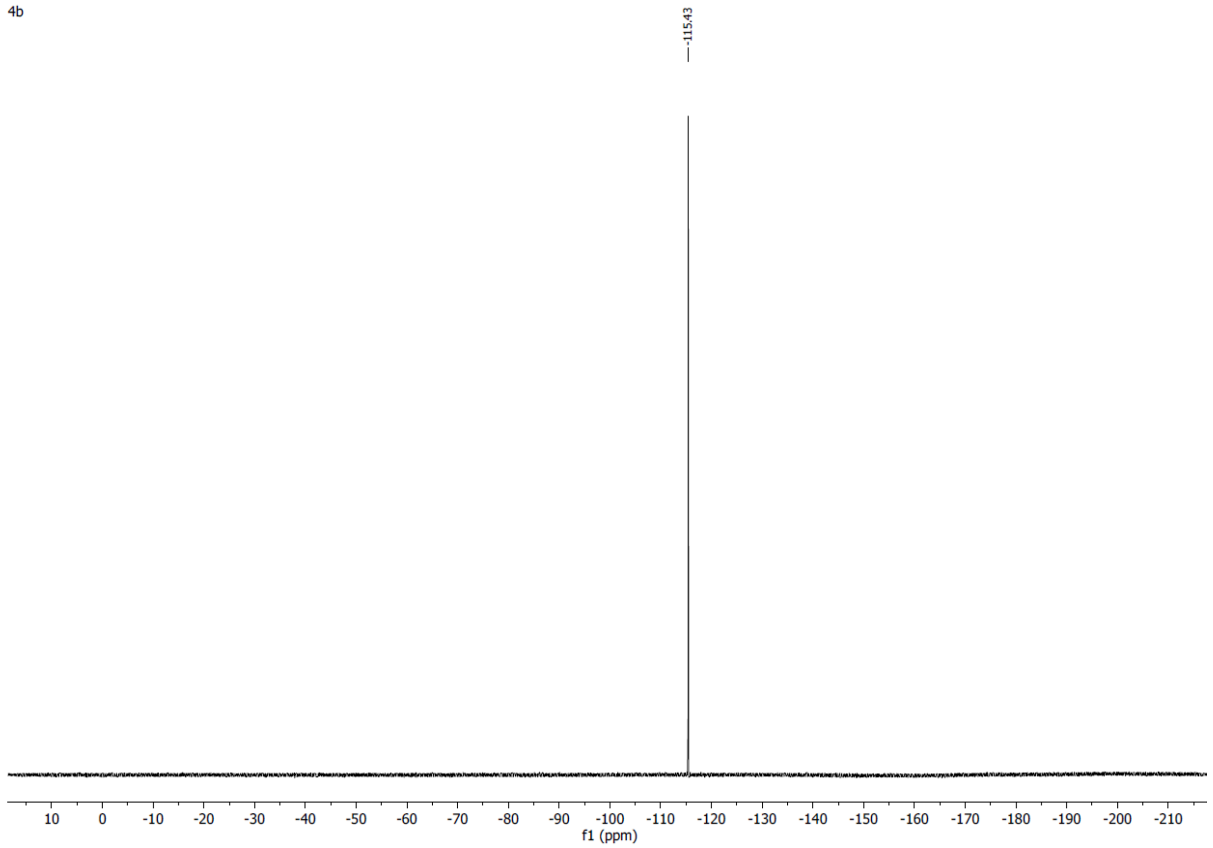

Figure S9. ^{19}F NMR (376 MHz, DMSO-*d*6) of **4b.**

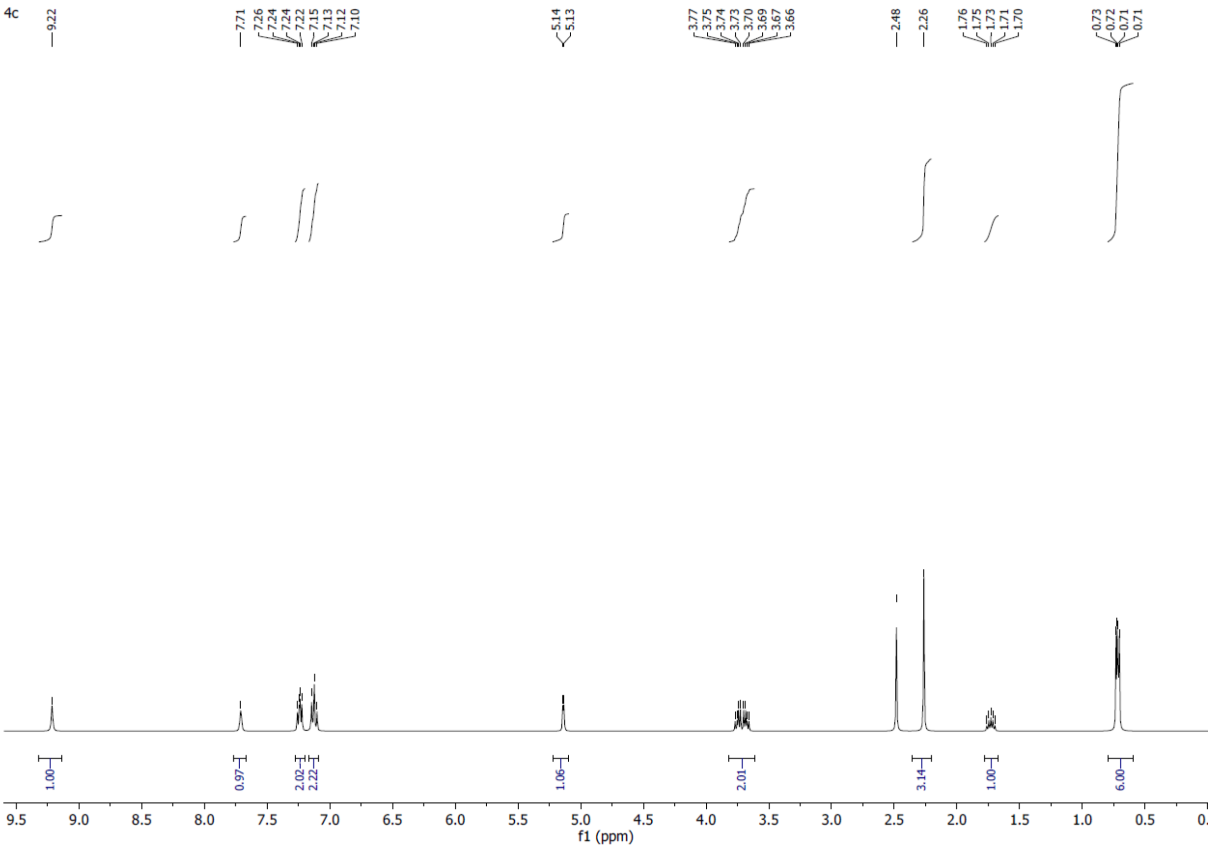

Figure S10. ^{1}H NMR (400 MHz, DMSO-*d6*) of **4c.**

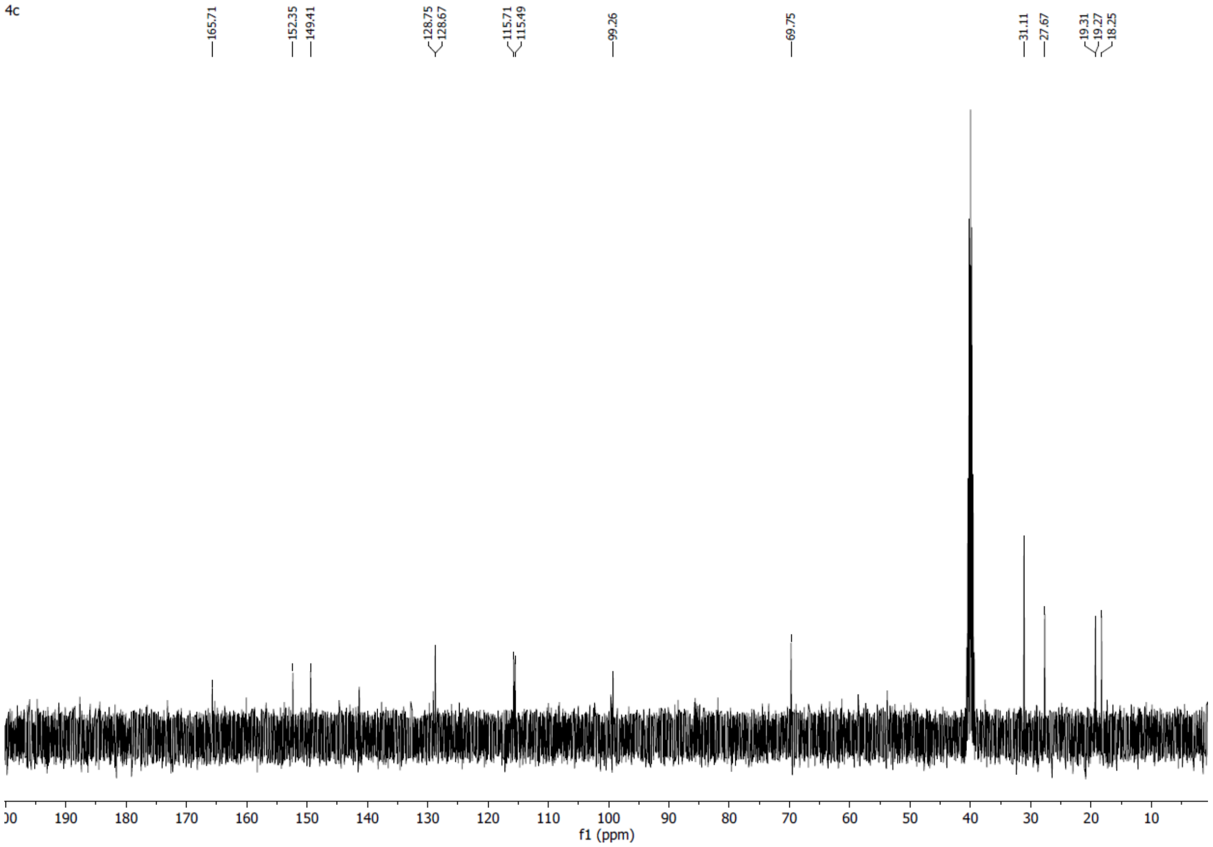

Figure S11. ^{13}C NMR (100 MHz, DMSO-*d6*) of **4c.**

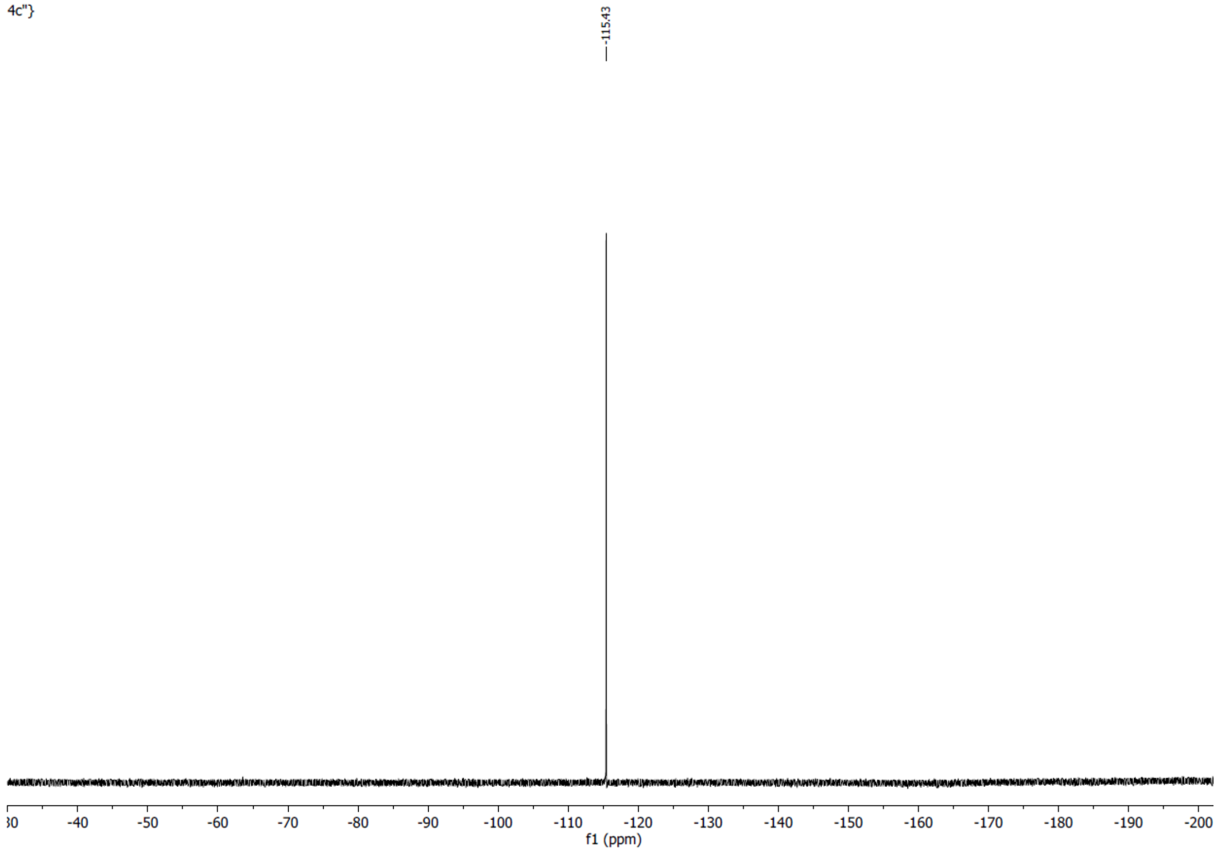

Figure 12. ^{19}F NMR (376 MHz, DMSO-*d*6) of **4c.**

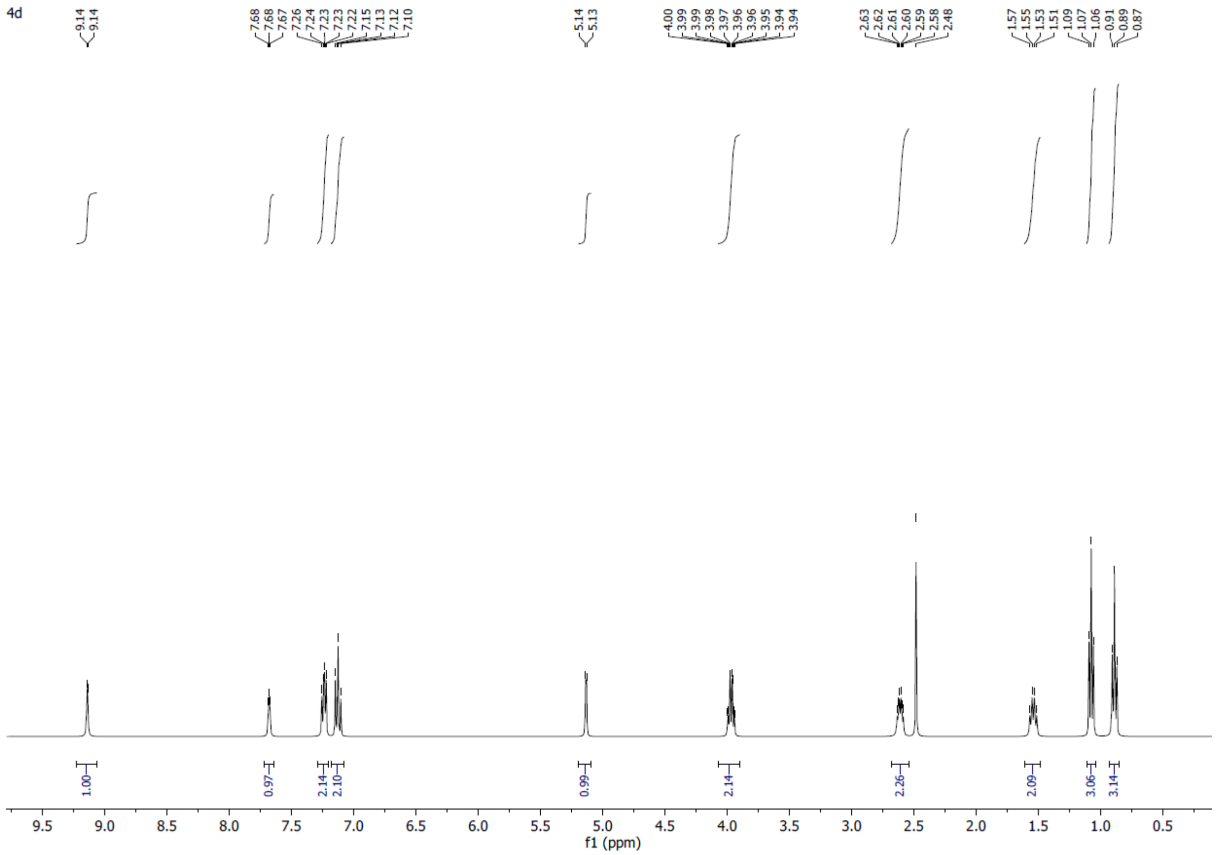

Figure S13. ^{1}H NMR (400 MHz, DMSO-*d*6) of **4d.**

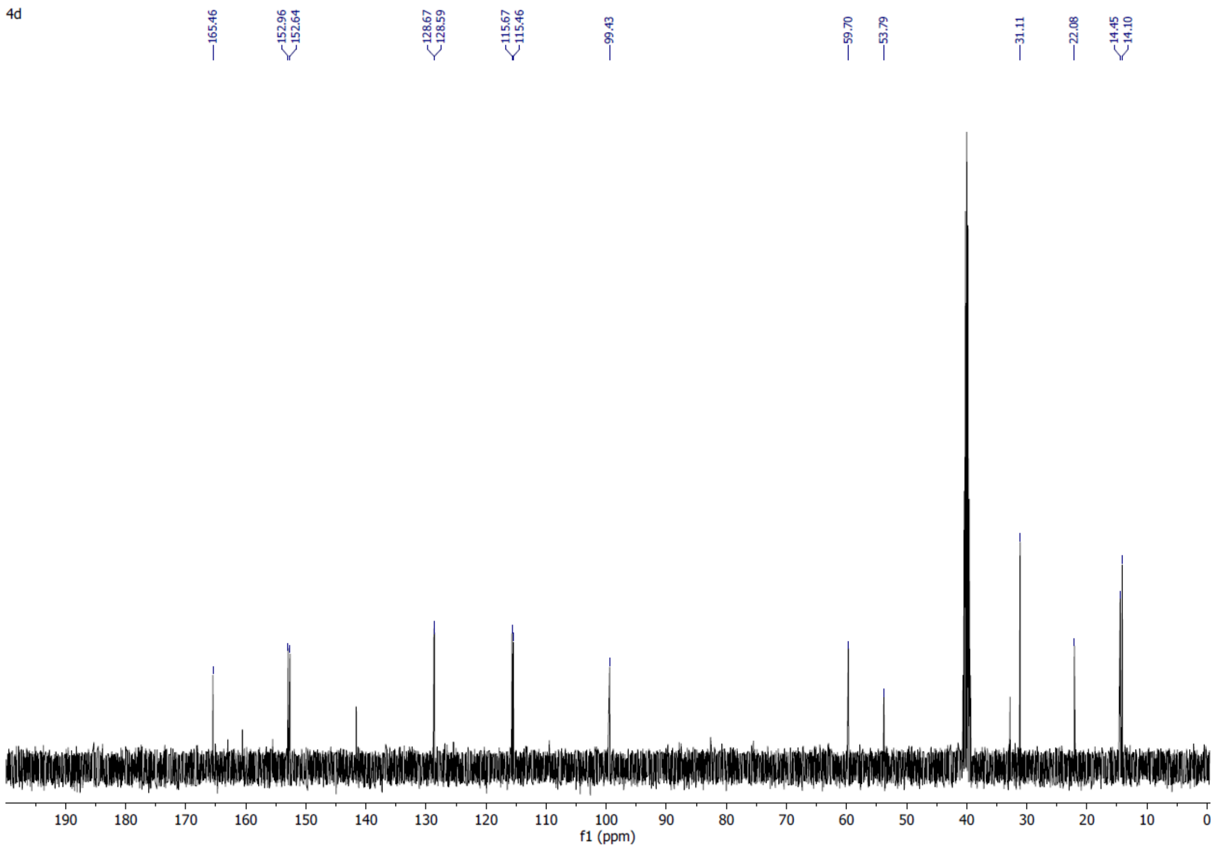

Figure S14. ^{13}C NMR (100 MHz, DMSO-*d6*) of **4d.**

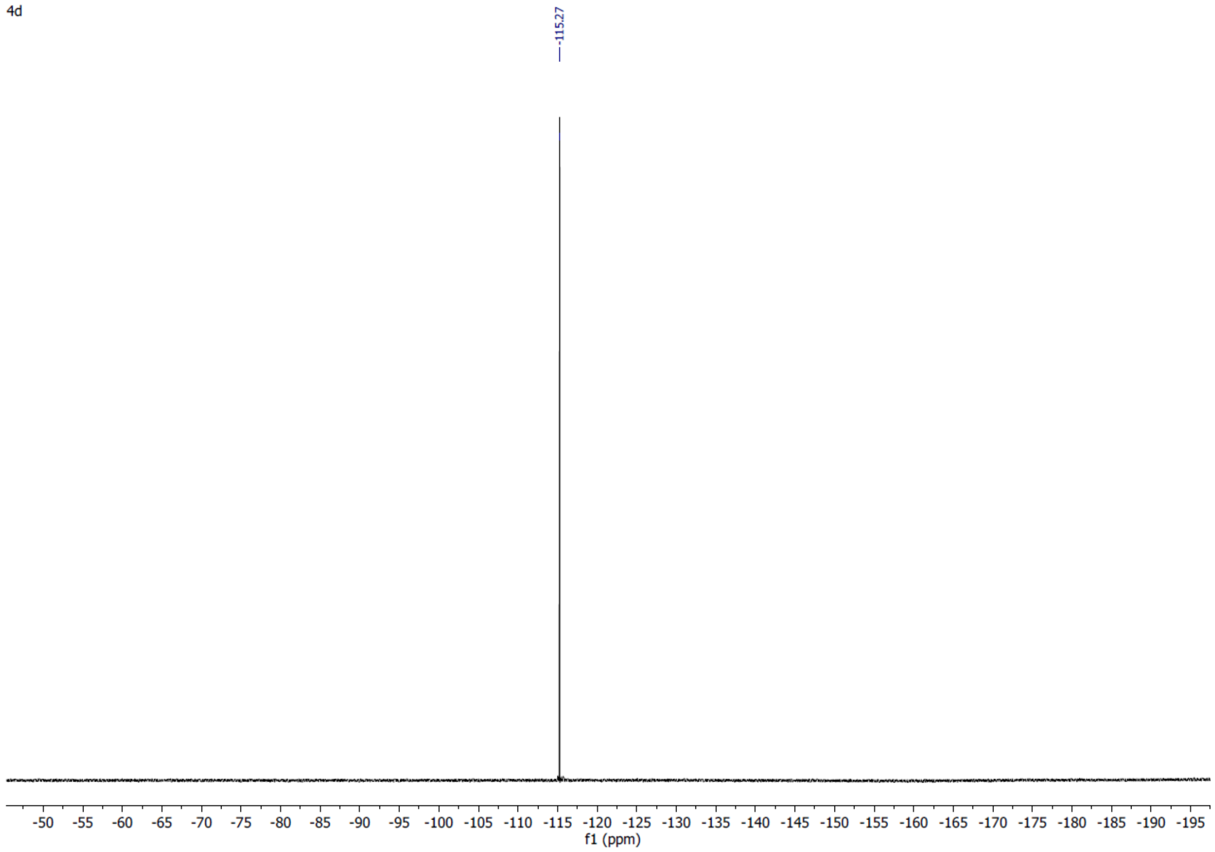

Figure S15. ^{19}F NMR (376 MHz, DMSO-*d6*) of **4d.**

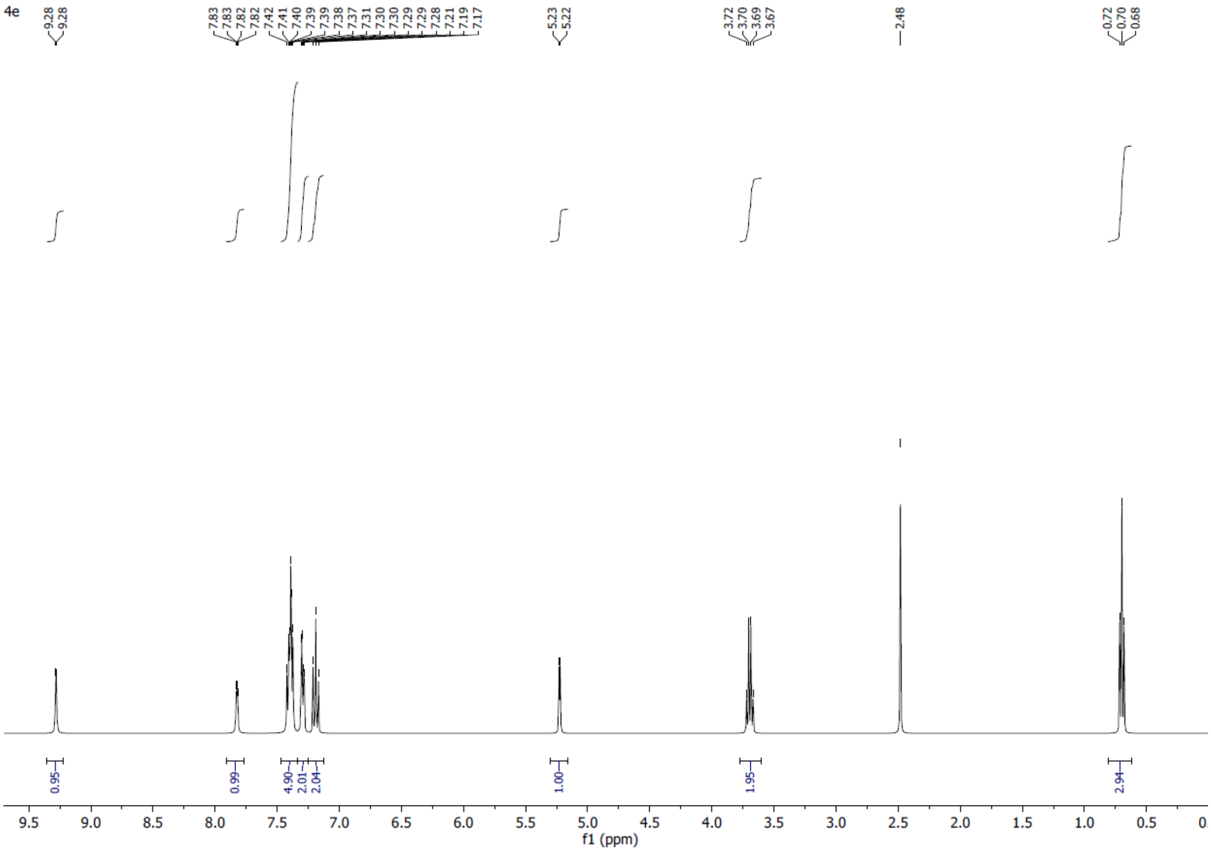

Figure S16. ^1H NMR (400 MHz, DMSO-*d6*) of **4e.**

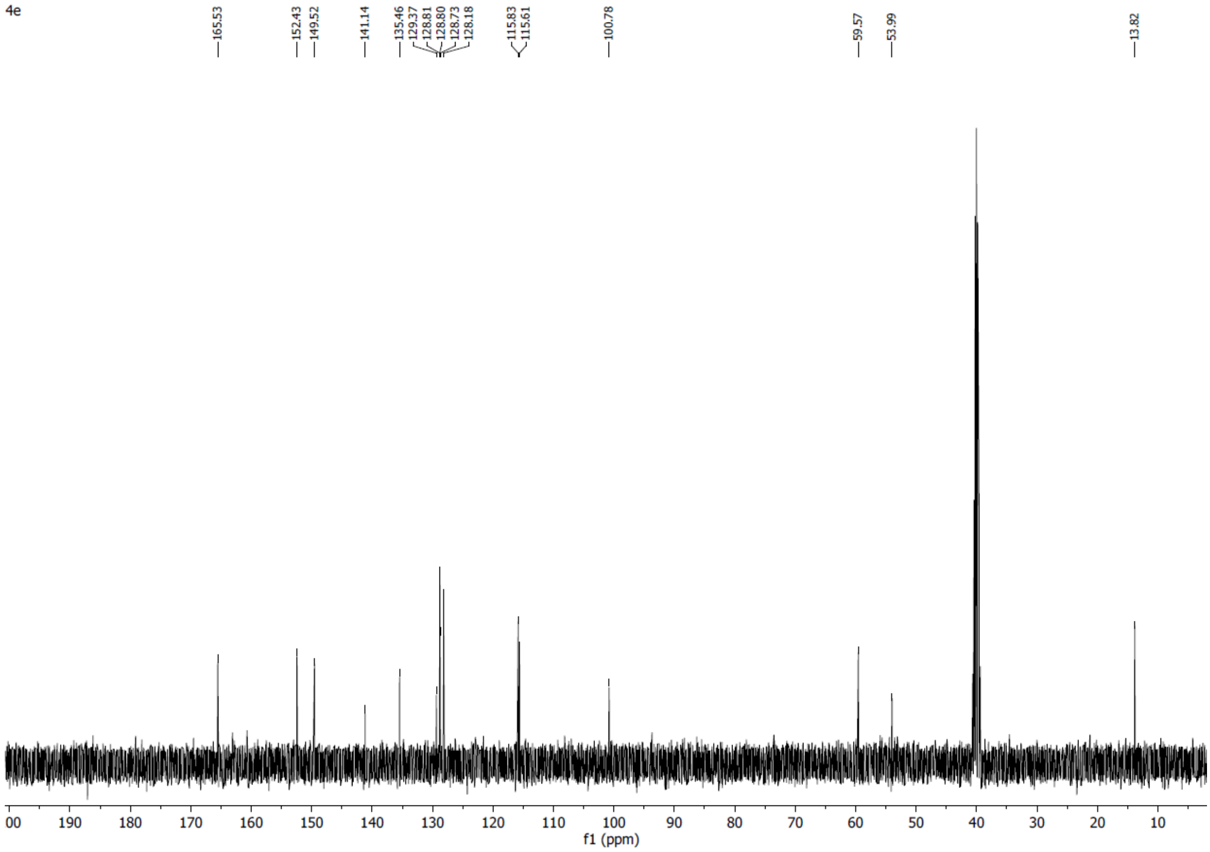

Figure S17. ^{13}C NMR (100 MHz, DMSO-*d6*) of **4e.**

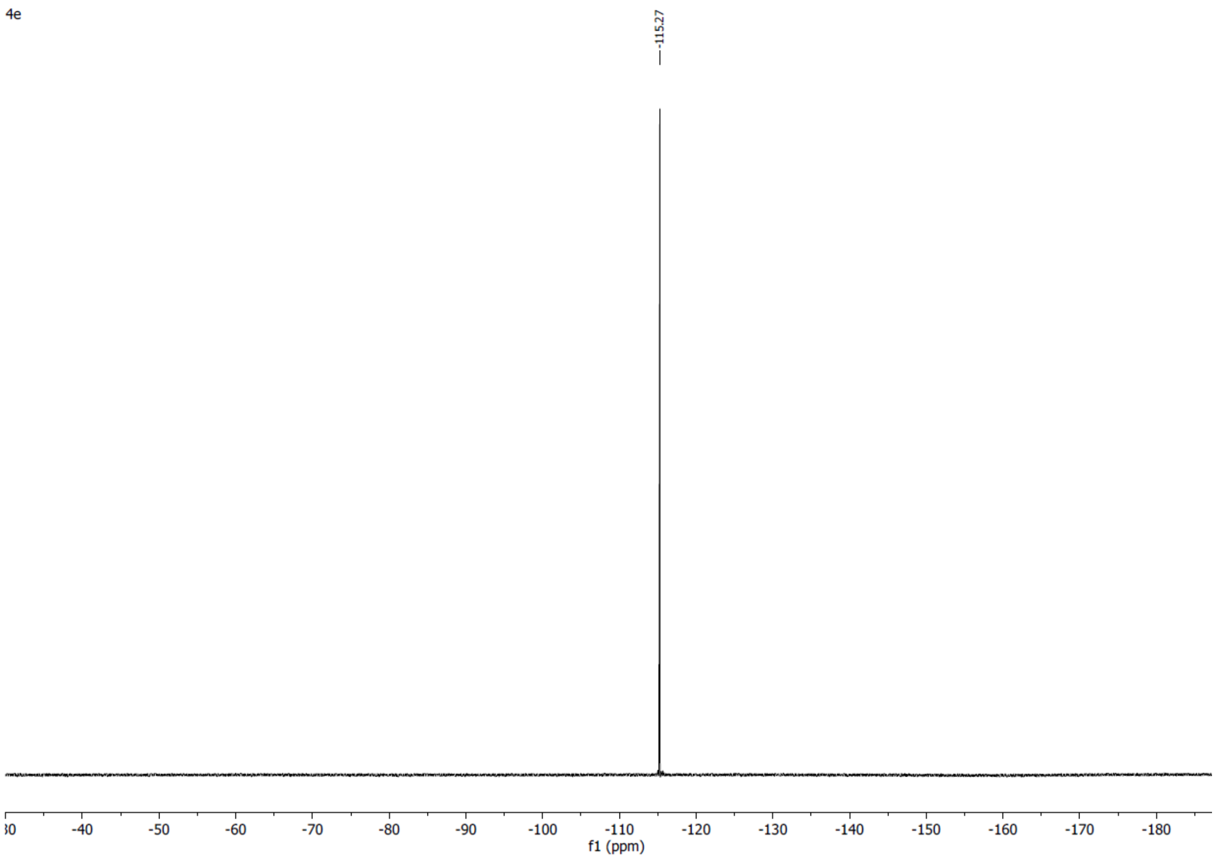

Figure S18. ^{19}F NMR (376 MHz, DMSO-*d*6) of **4e.**

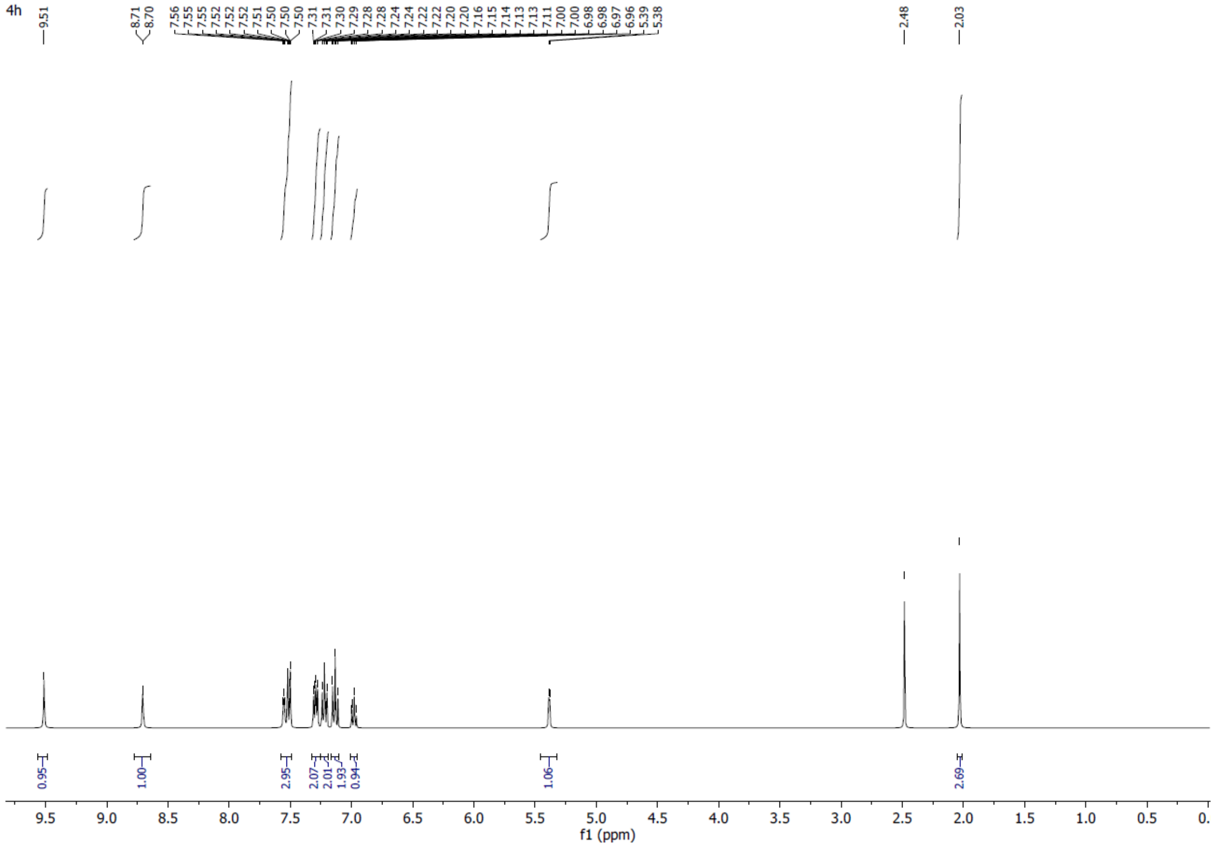

Figure S19. ^{1}H NMR (400 MHz, DMSO-*d*6) of **4h.**

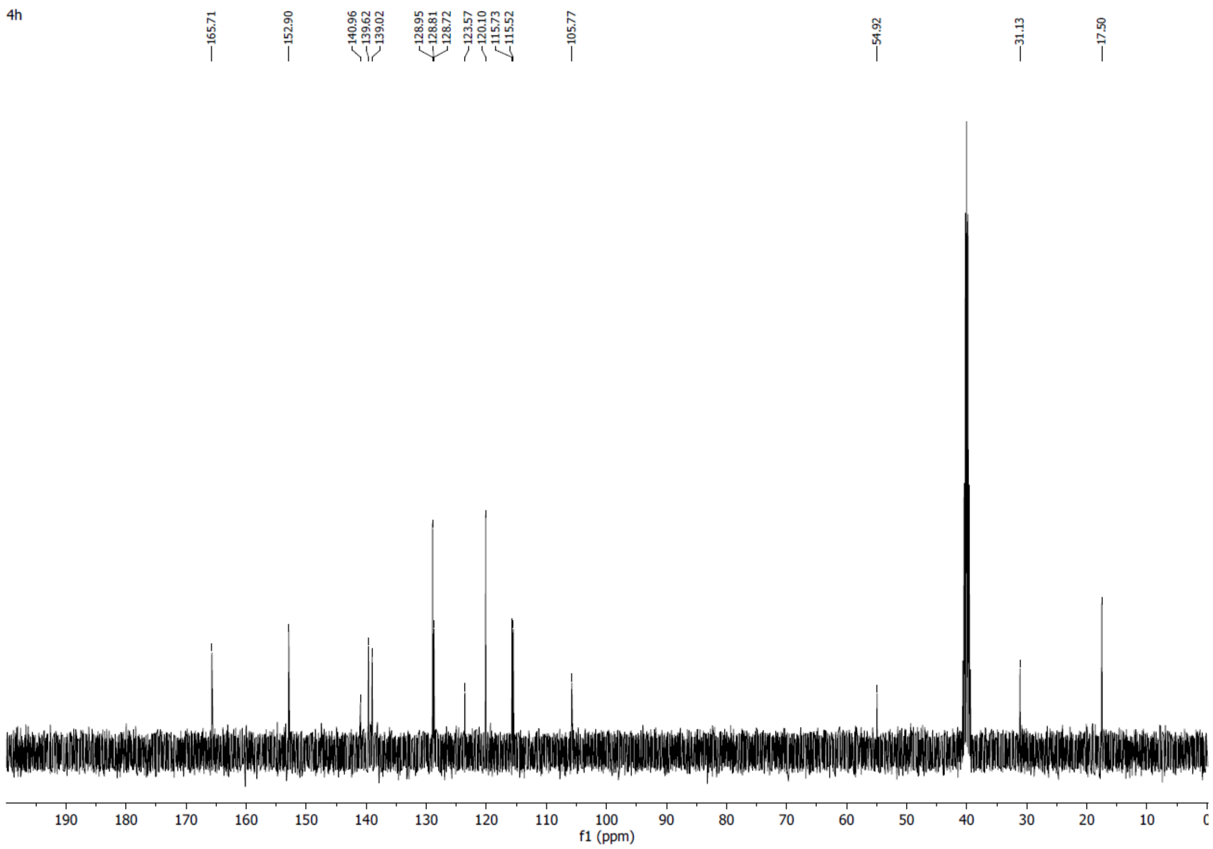

Figure S20. ^{13}C NMR (100 MHz, DMSO-*d6*) of **4h.**

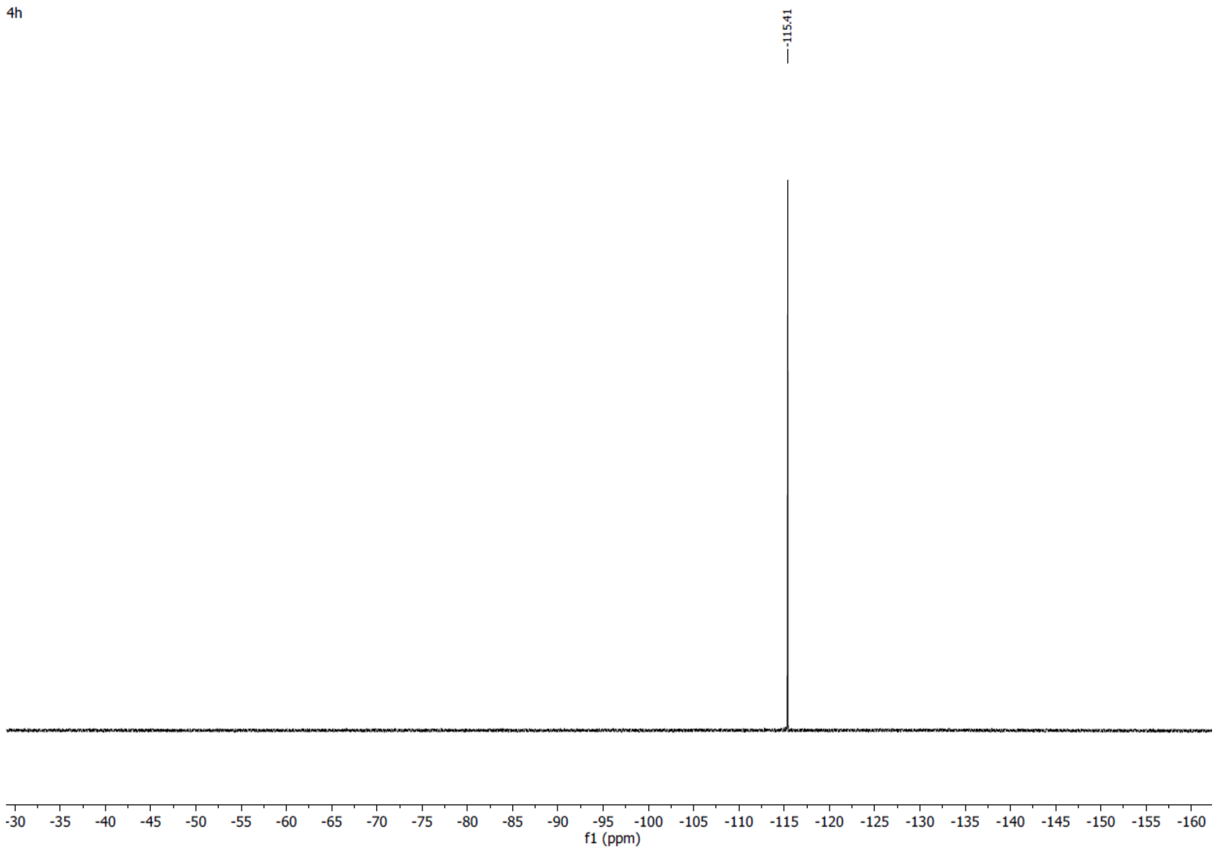

Figure S21. ^{19}F NMR (376 MHz, DMSO-*d6*) of **4h.**

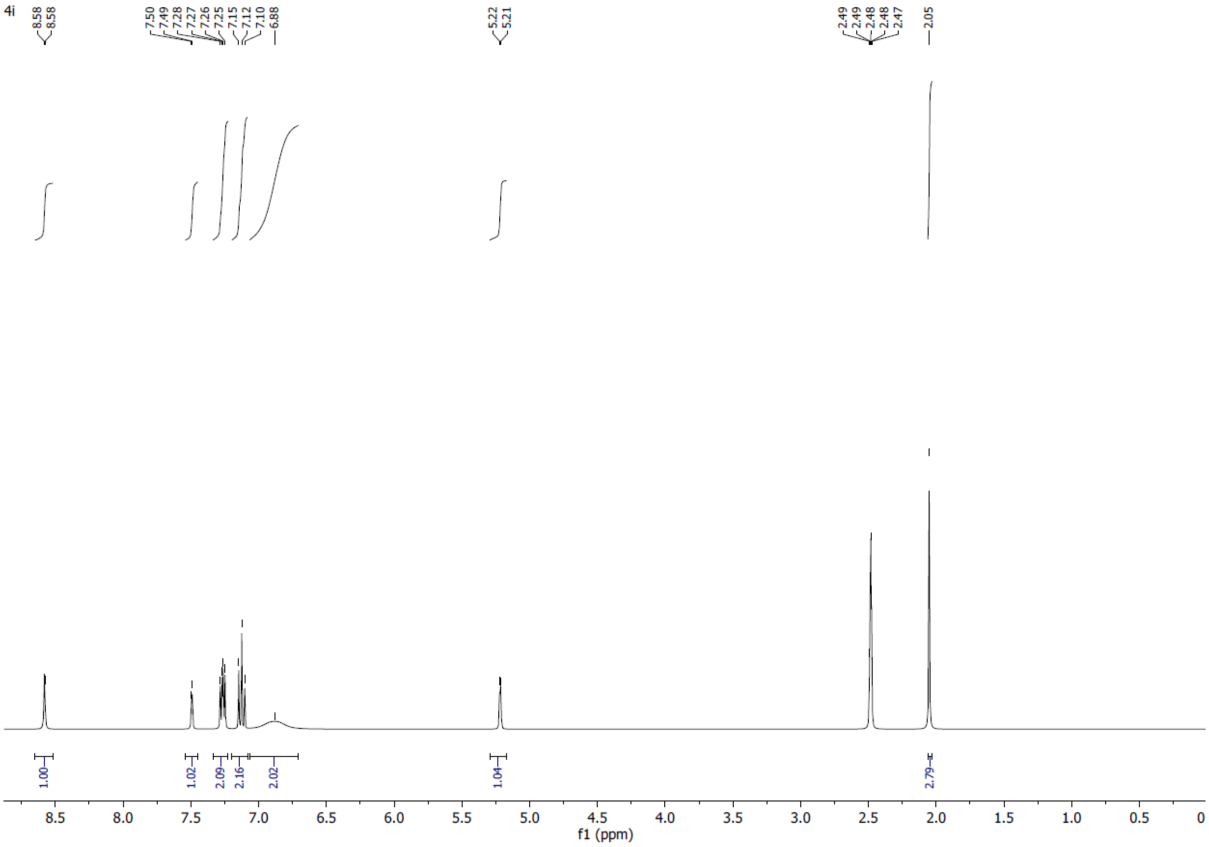

Figure S22. ^{1}H NMR (400 MHz, DMSO-*d6*) of **4i.**

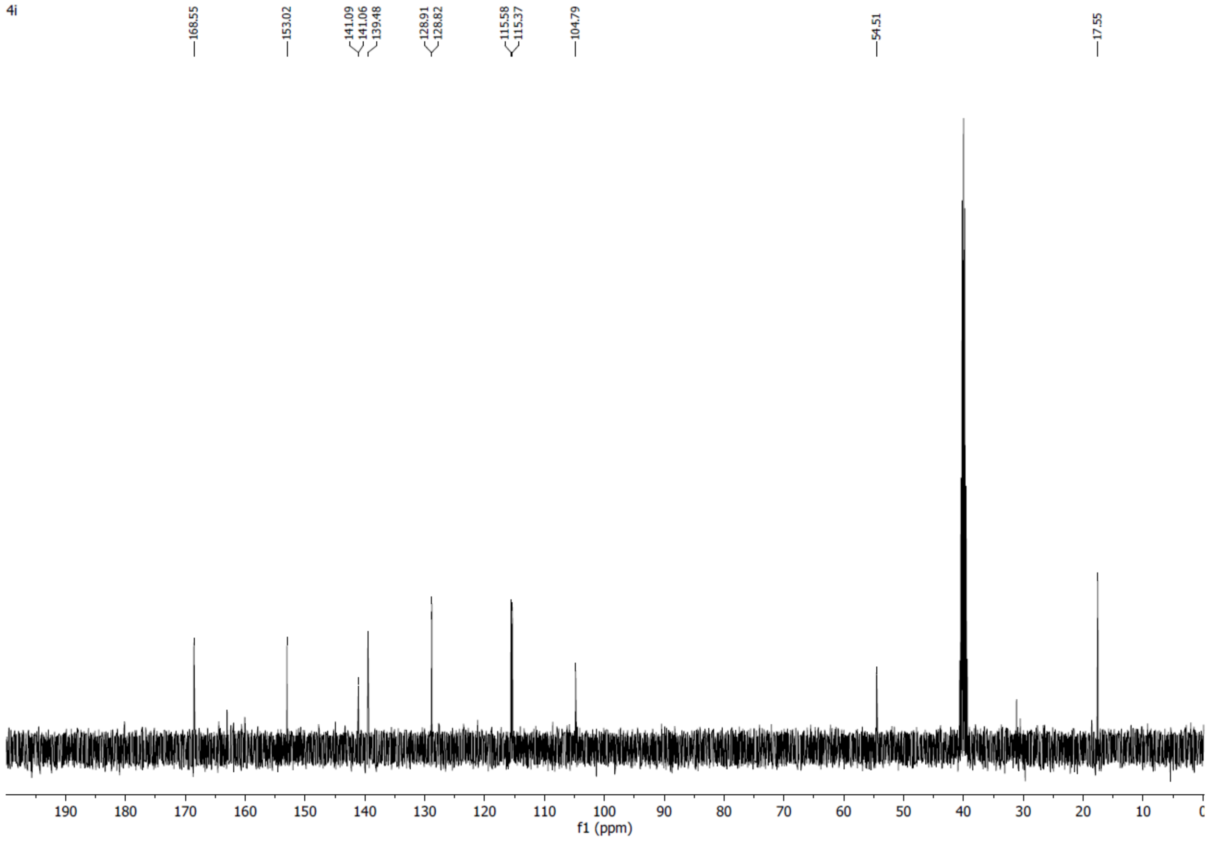

Figure S23. ^{13}C NMR (100 MHz, DMSO-*d6*) of **4i.**

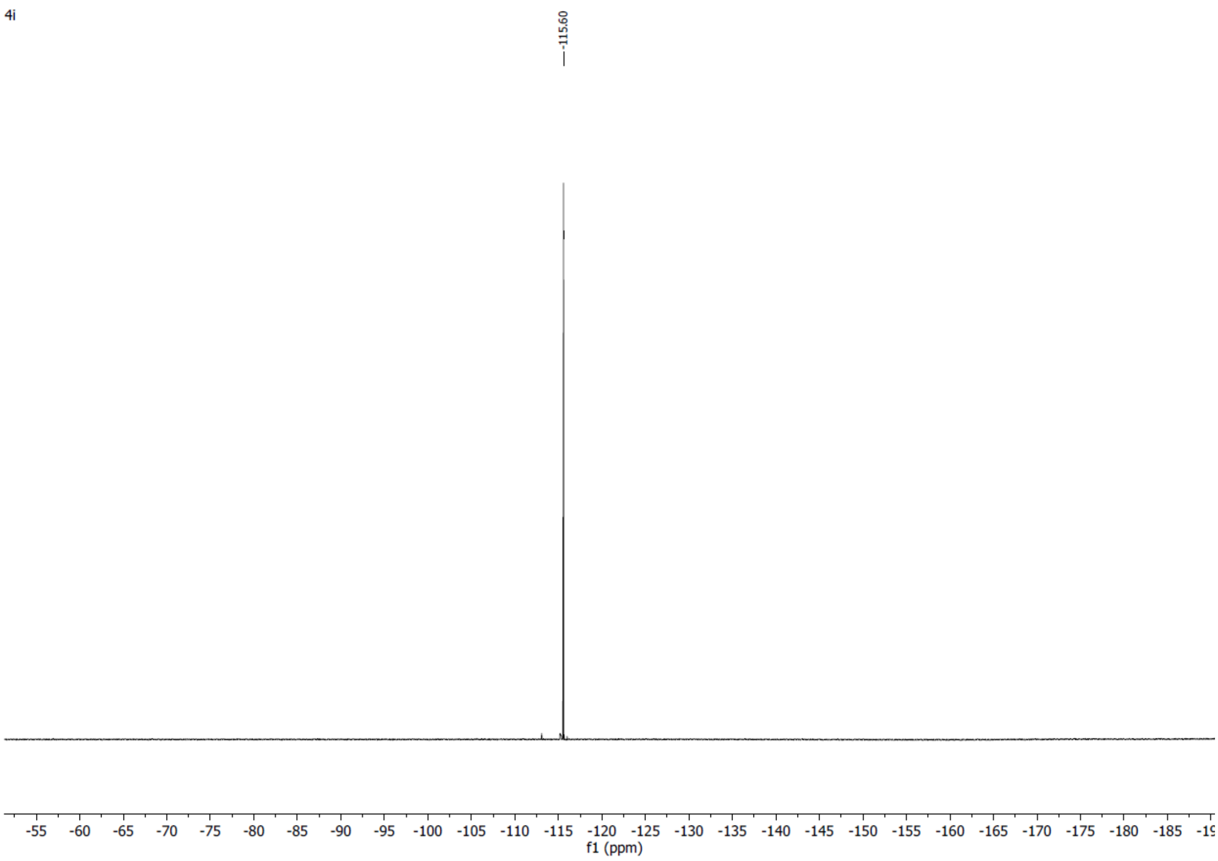

Figure S24. ^{19}F NMR (376 MHz, DMSO-*d*6) of **4i.**

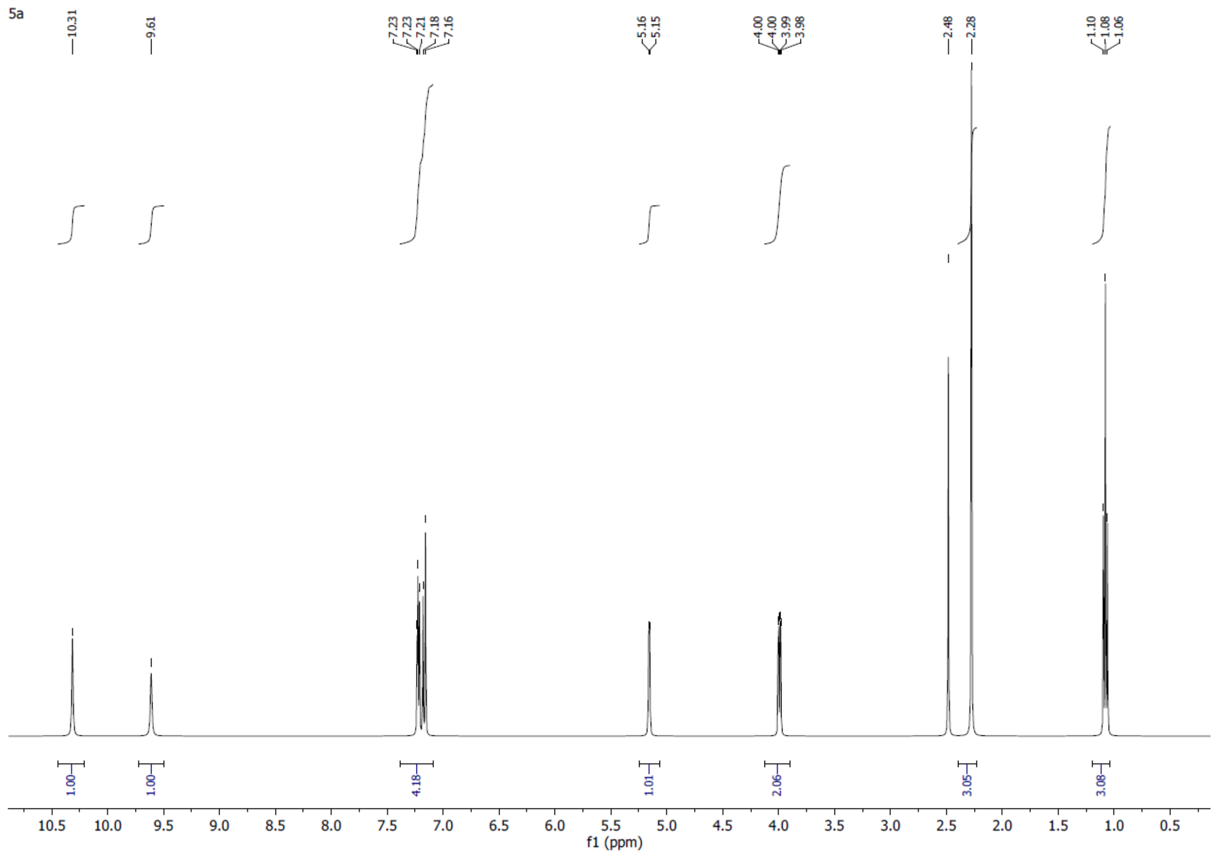

Figure S25. ^{1}H NMR (400 MHz, DMSO-*d*6) of **5a.**

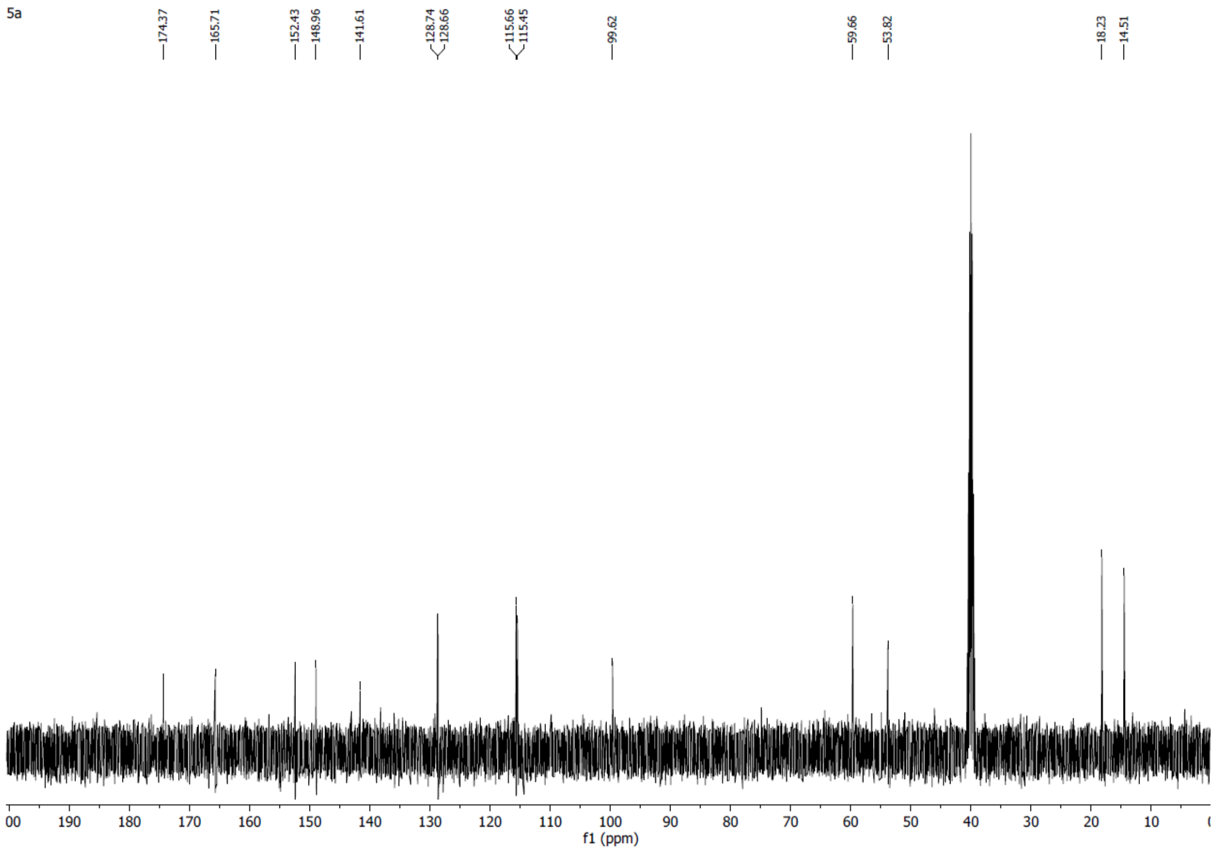

Figure S26. ^{13}C NMR (100 MHz, DMSO-*d*6) of **5a.**

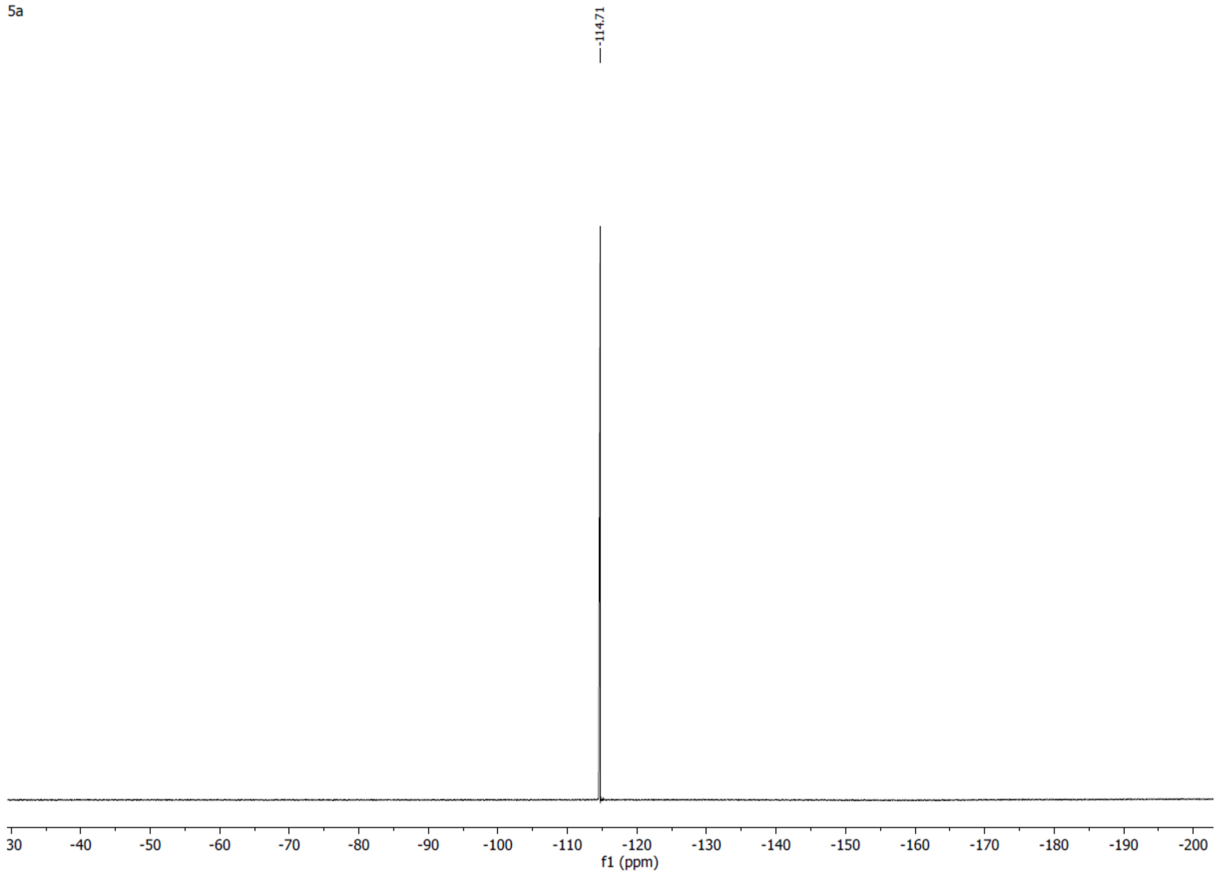

Figure S27. ^{19}F NMR (376 MHz, DMSO-*d*6) of **5a.**

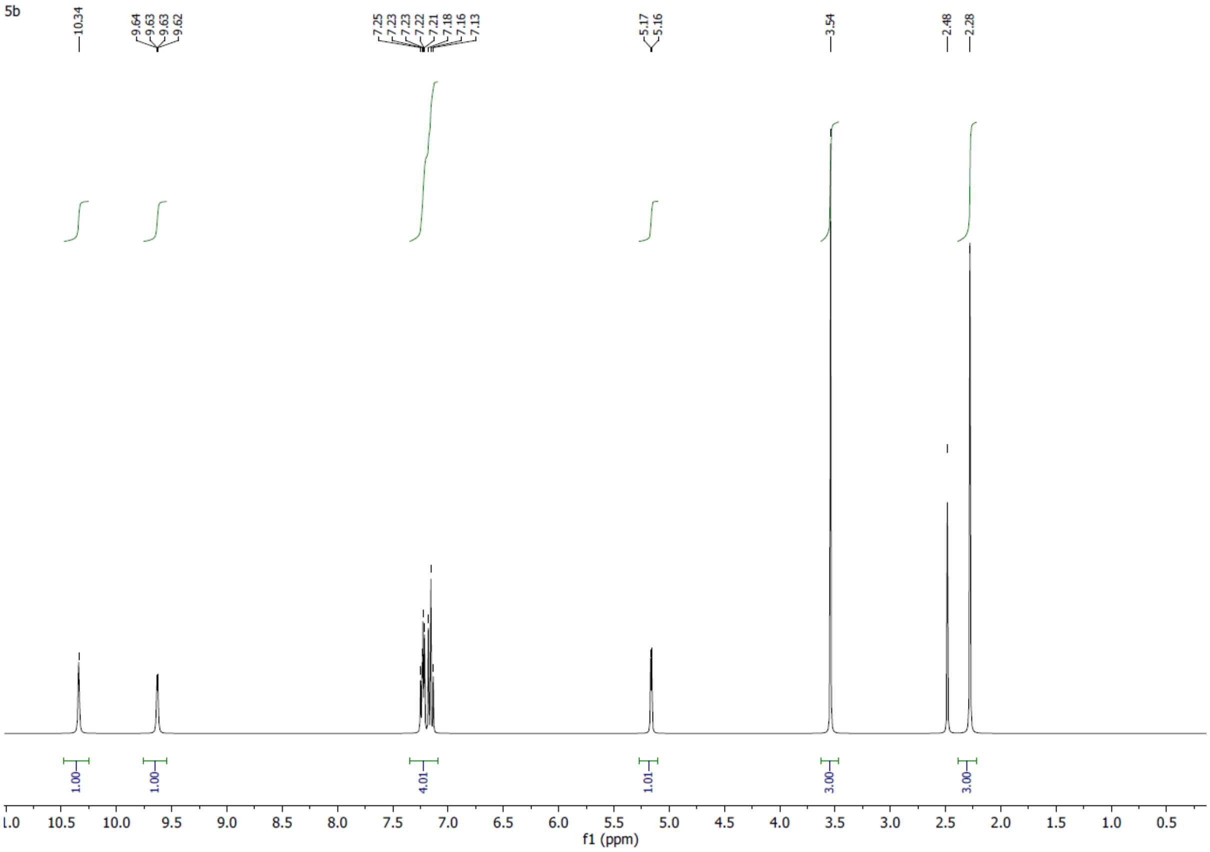

Figure S28. ^{1}H NMR (400 MHz, DMSO-*d*6) of **5b.**

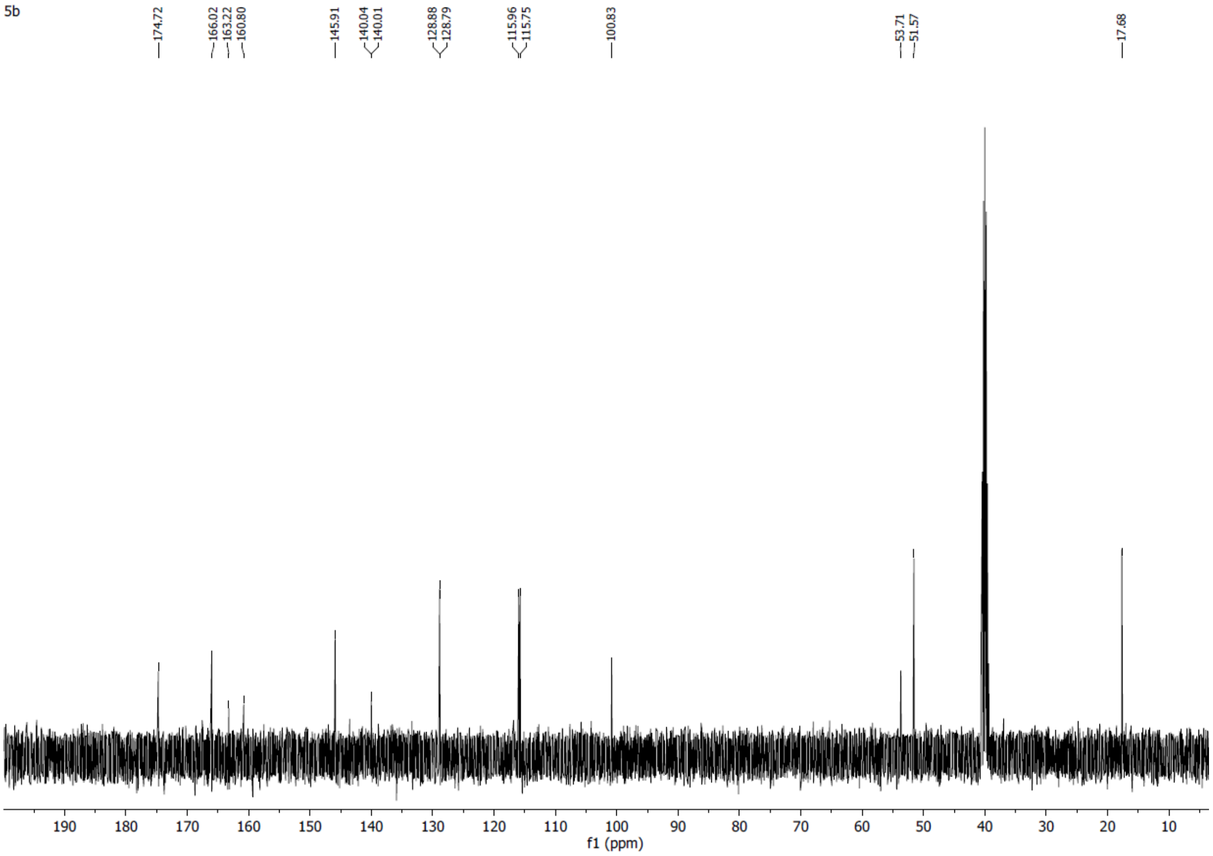

Figure S29. ^{13}C NMR (100 MHz, DMSO-*d*6) of **5b.**

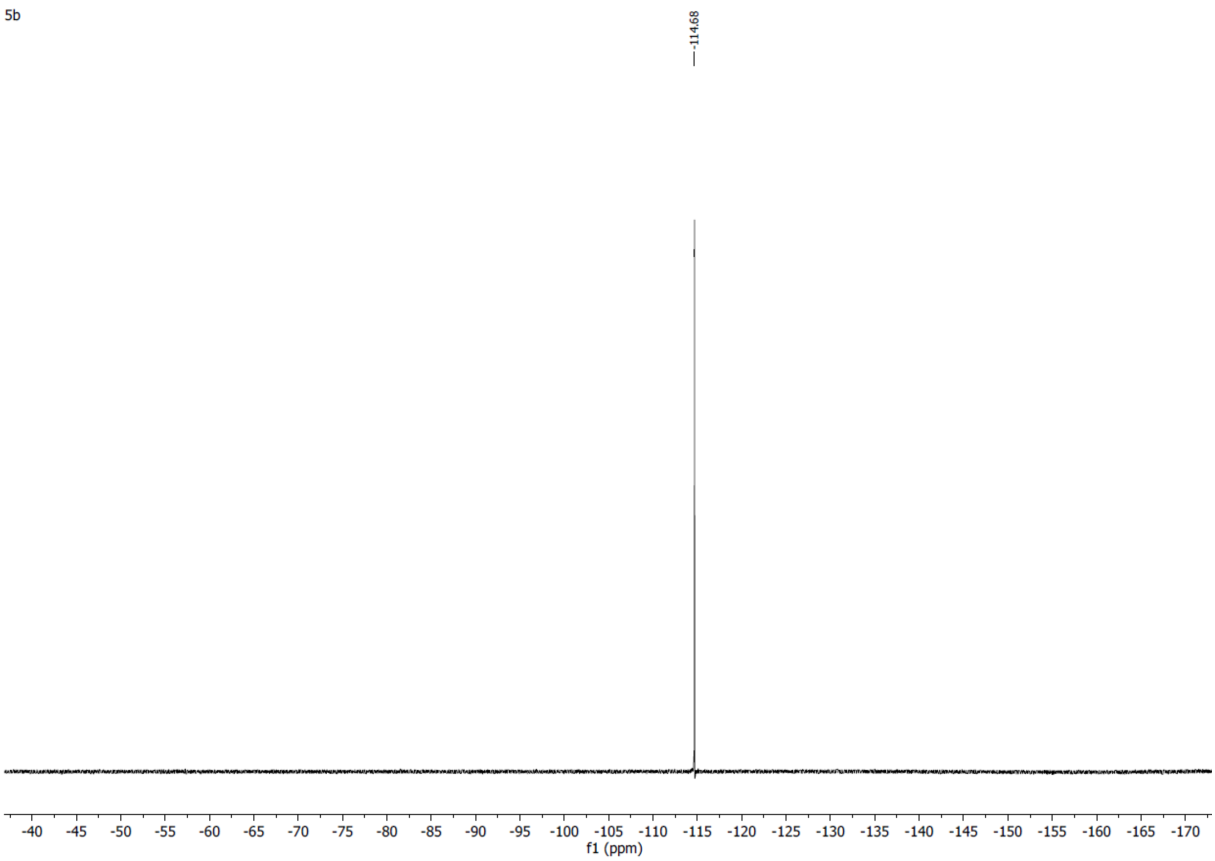

Figure S30. ^{19}F NMR (376 MHz, DMSO-*d*6) of **5b.**

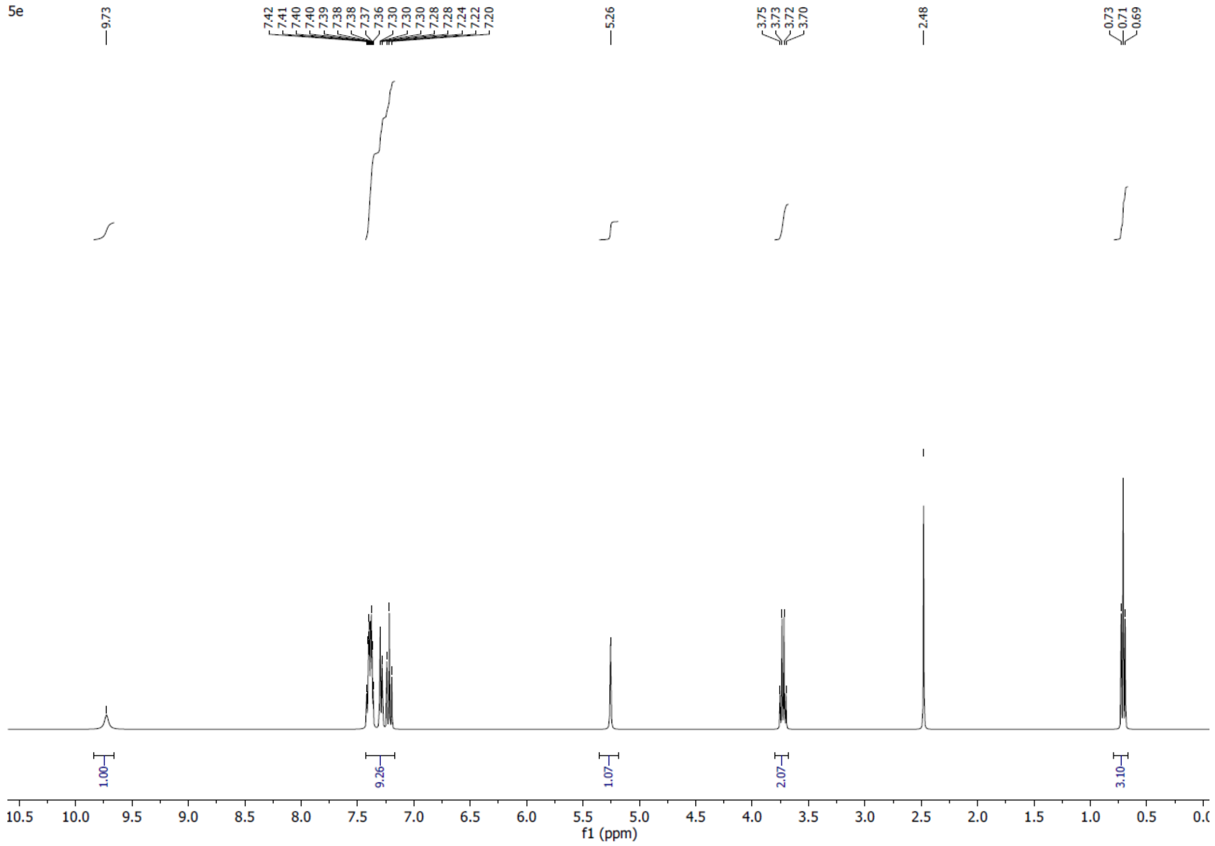

Figure S31. ^{1}H NMR (400 MHz, DMSO-*d*6) of **5e.**

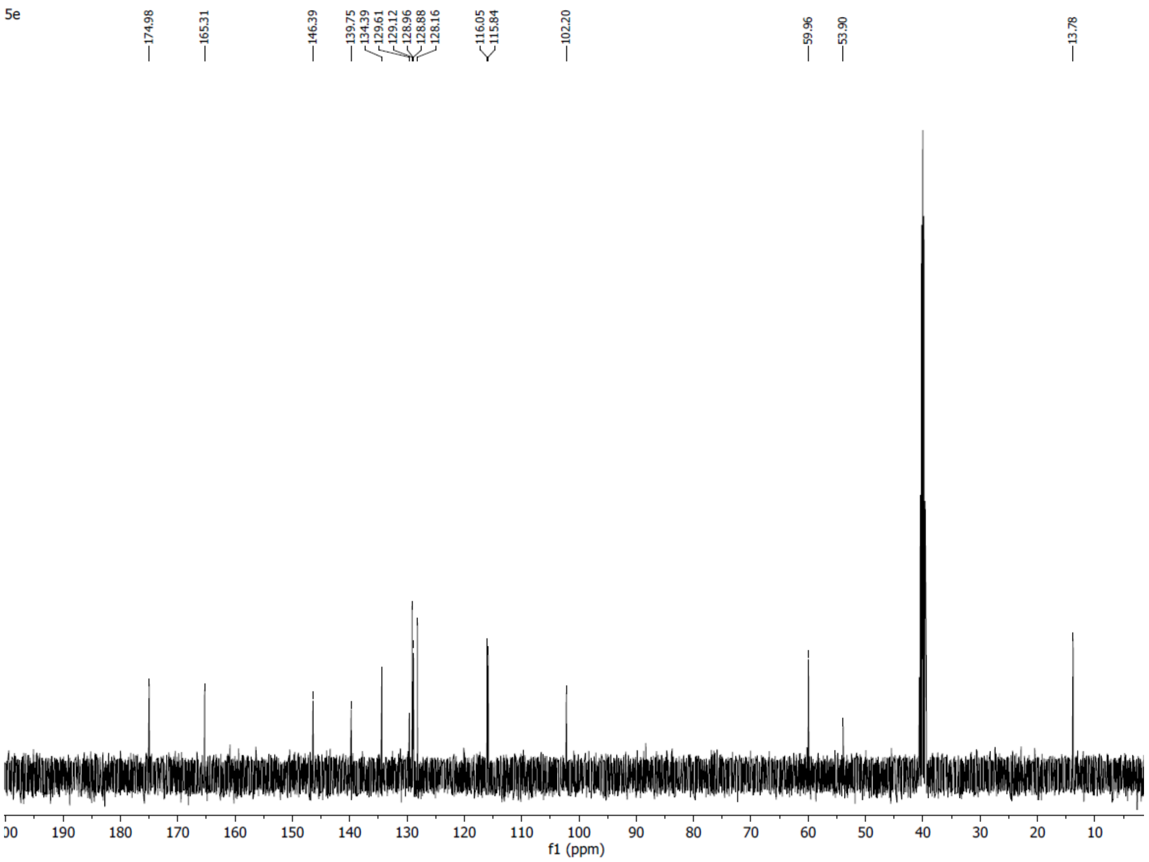

Figure S32. ^{13}C NMR (100 MHz, DMSO-*d6*) of **5e.**

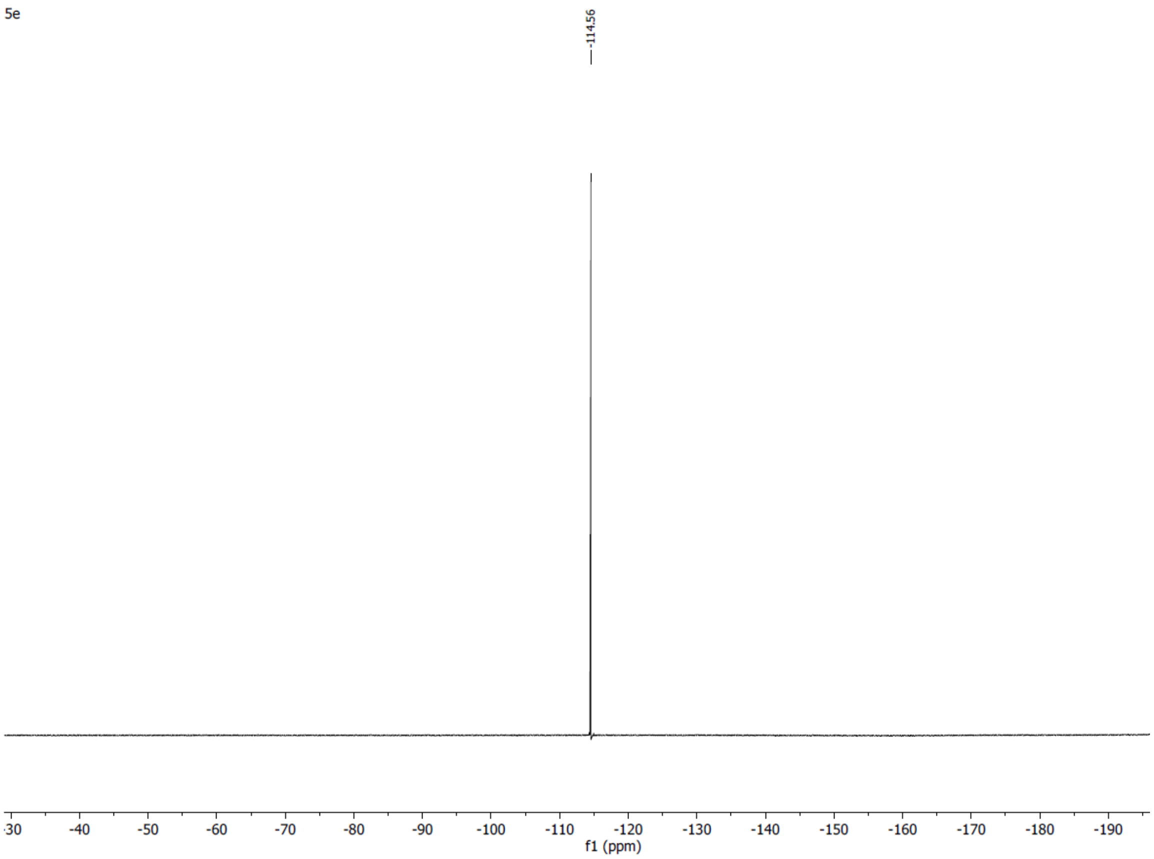

Figure S33. ^{19}F NMR (376 MHz, DMSO-*d6*) of **5e.**

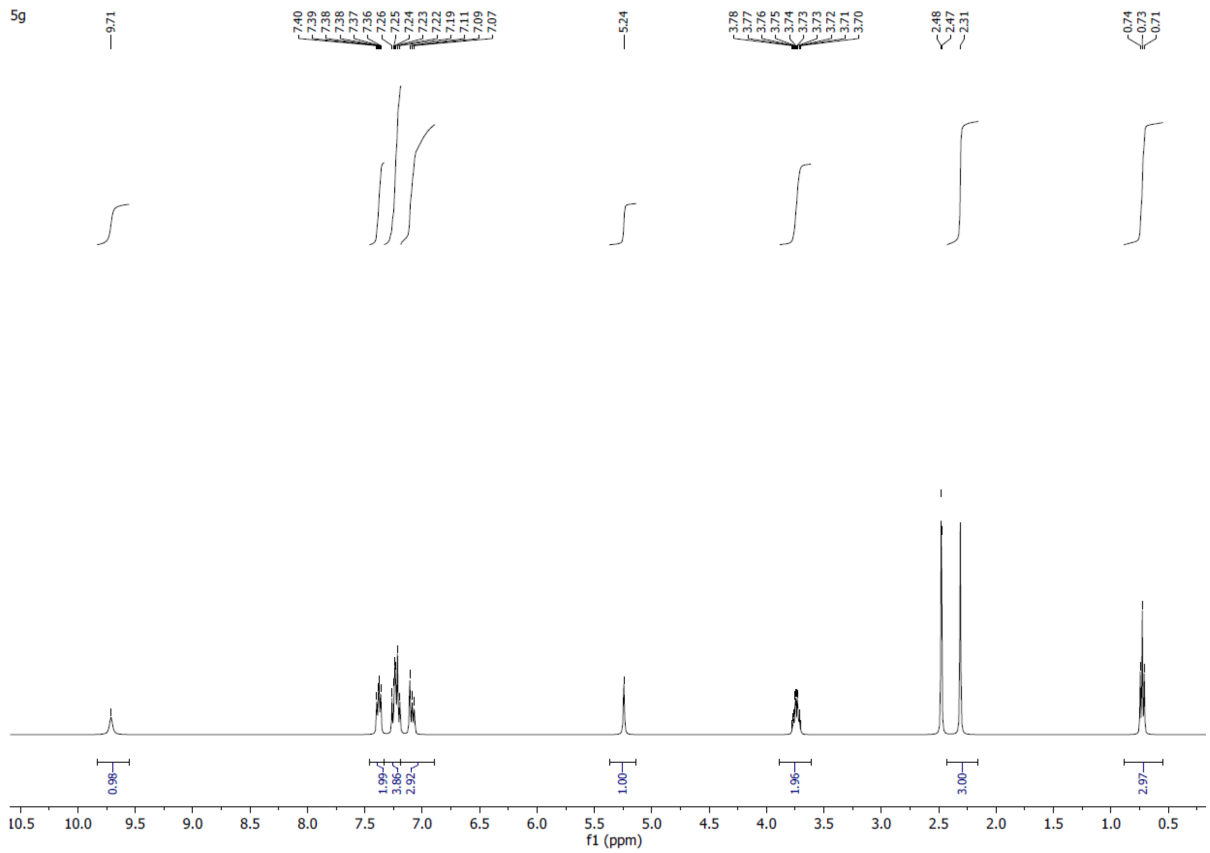

Figure S34. ^{1}H NMR (400 MHz, DMSO-*d*6) of **5g.**

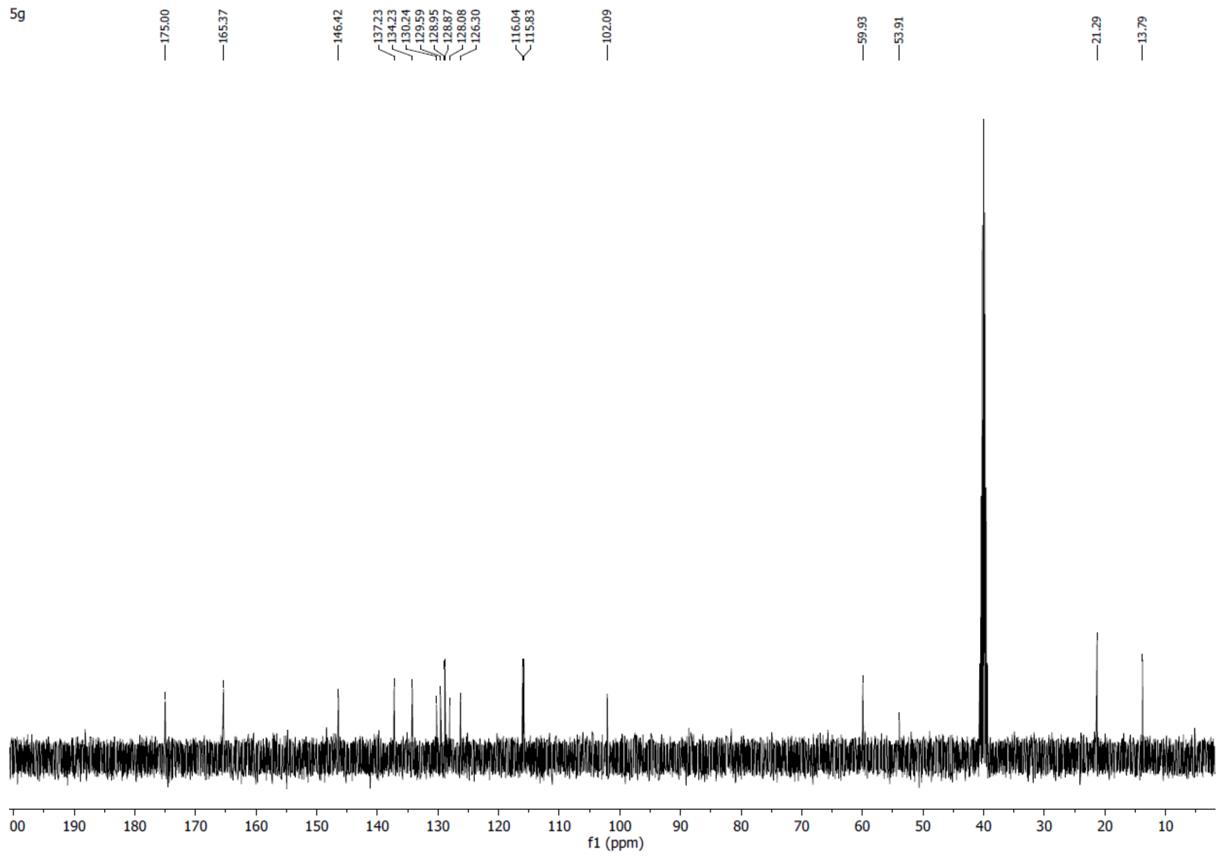

Figure S35. ^{13}C NMR (100 MHz, DMSO-*d*6) of **5g.**

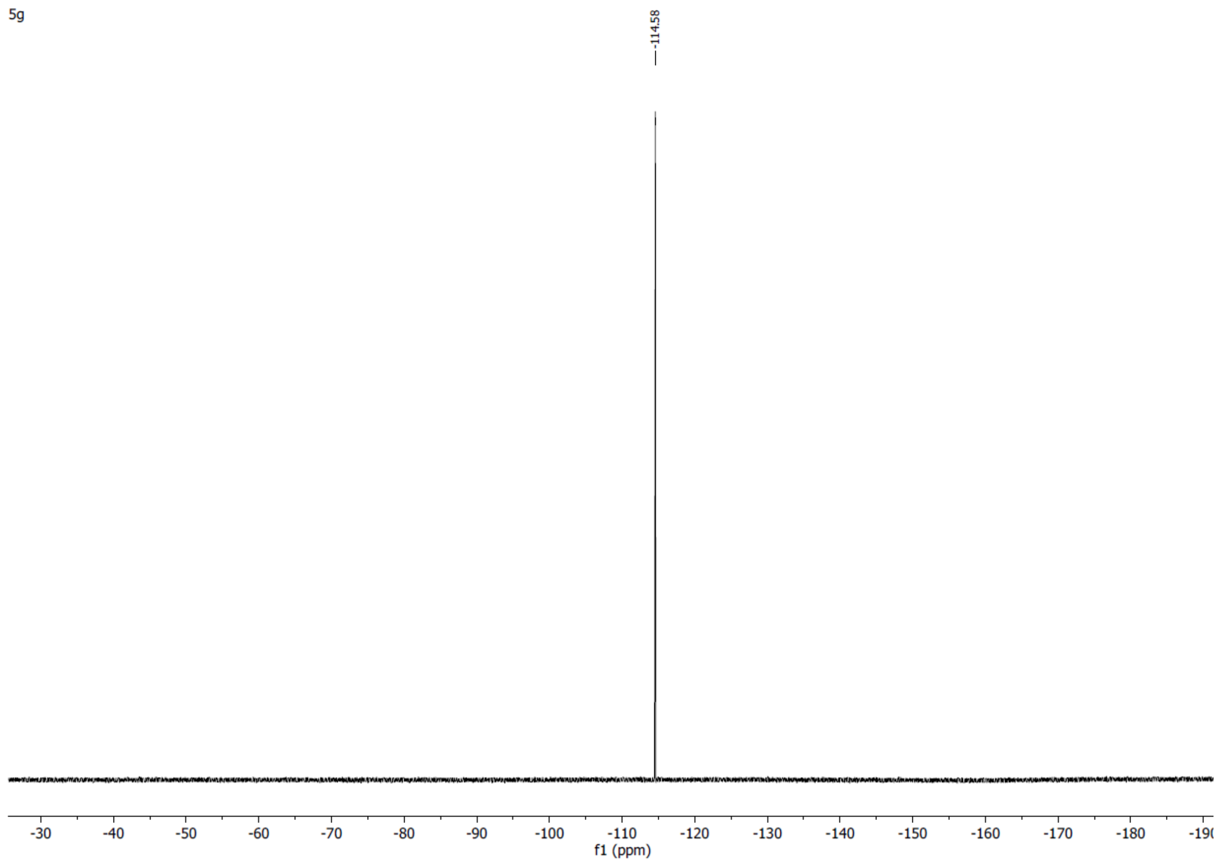

Figure S36. ^{19}F NMR (376 MHz, DMSO-*d6*) of **5g.**

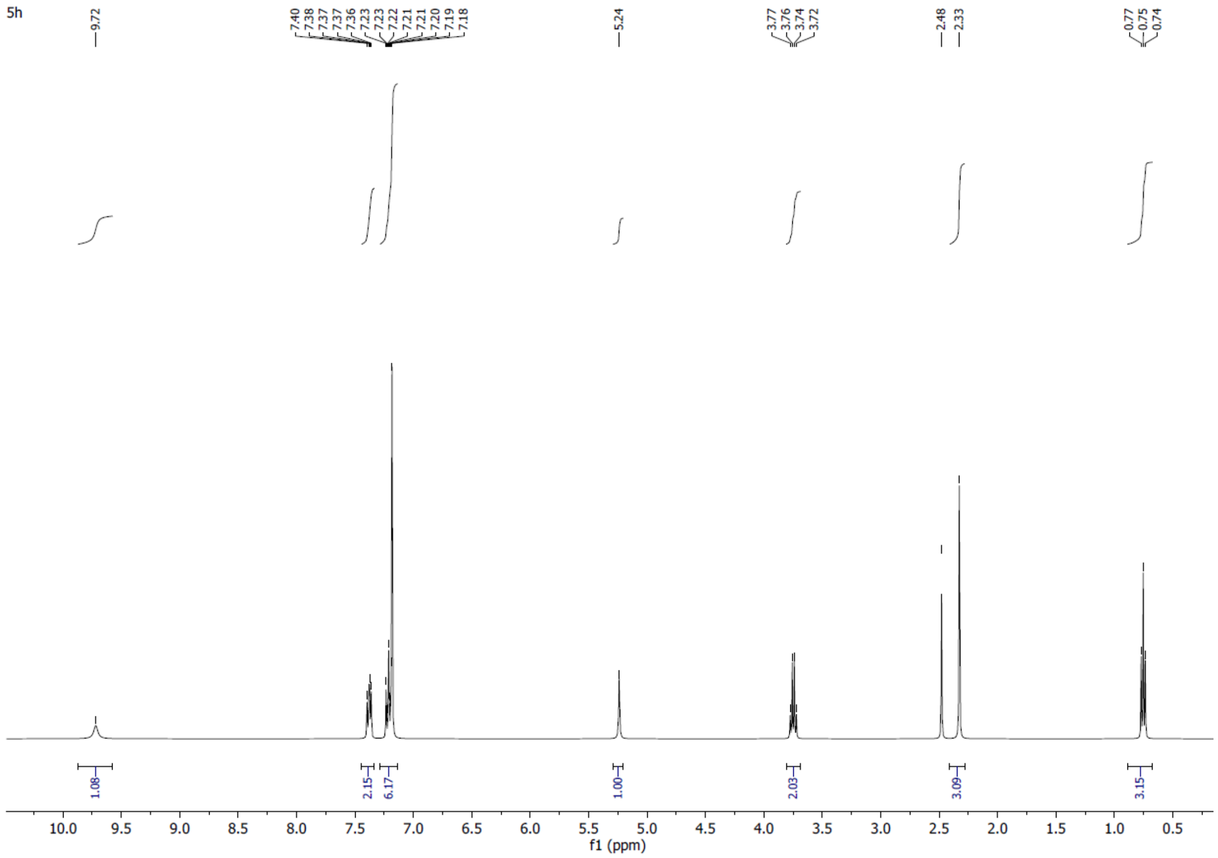

Figure S37. ^{1}H NMR (400 MHz, DMSO-*d6*) of **5h.**

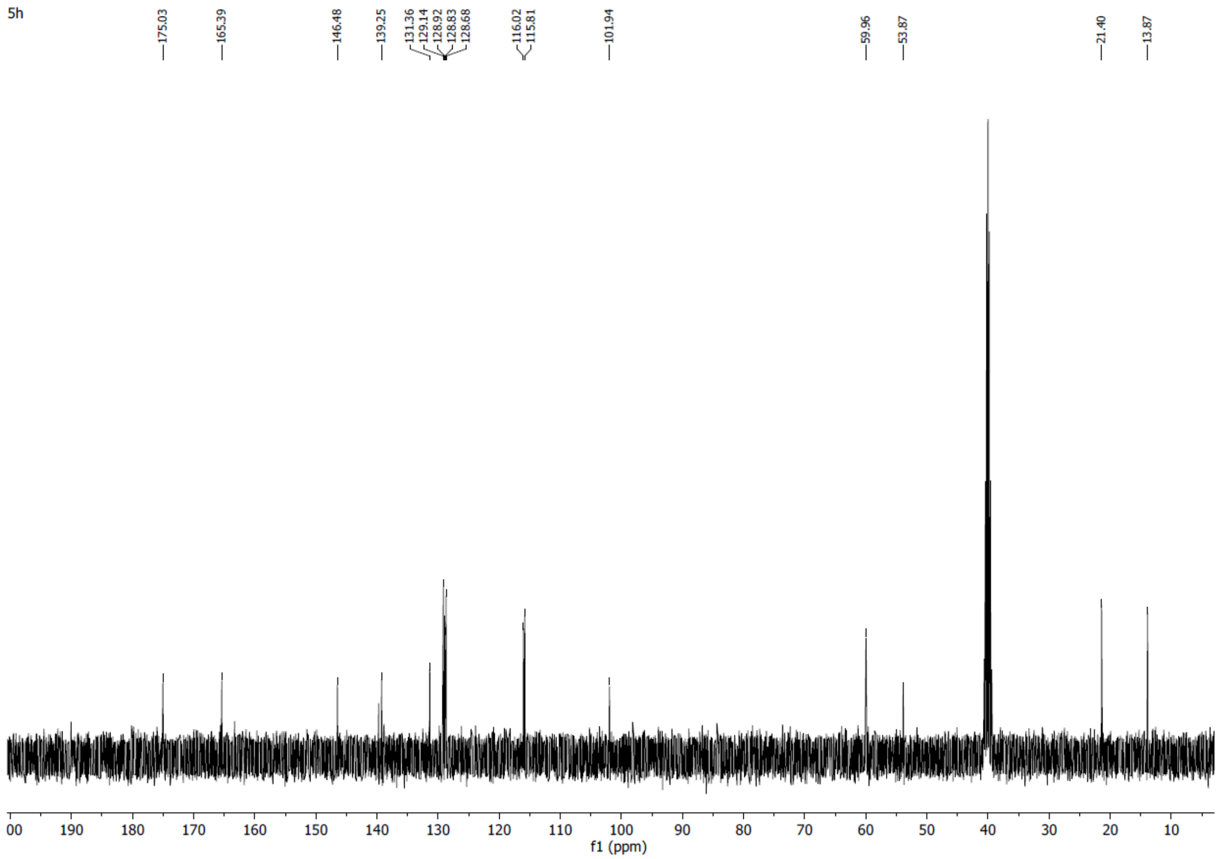

Figure S38. ^{13}C NMR (100 MHz, DMSO-*d*6) of **5h.**

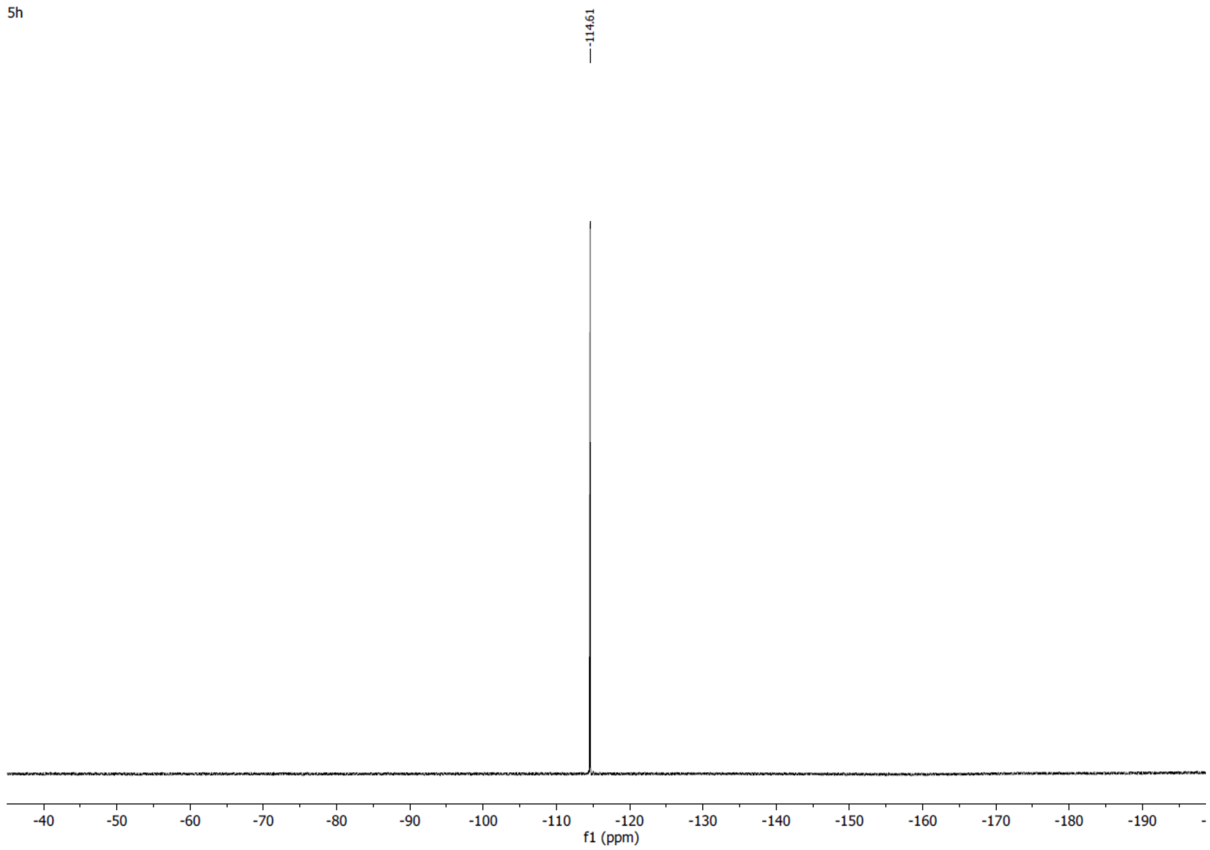

Figure S39. ^{19}F NMR (376 MHz, DMSO-*d*6) of **5h.**

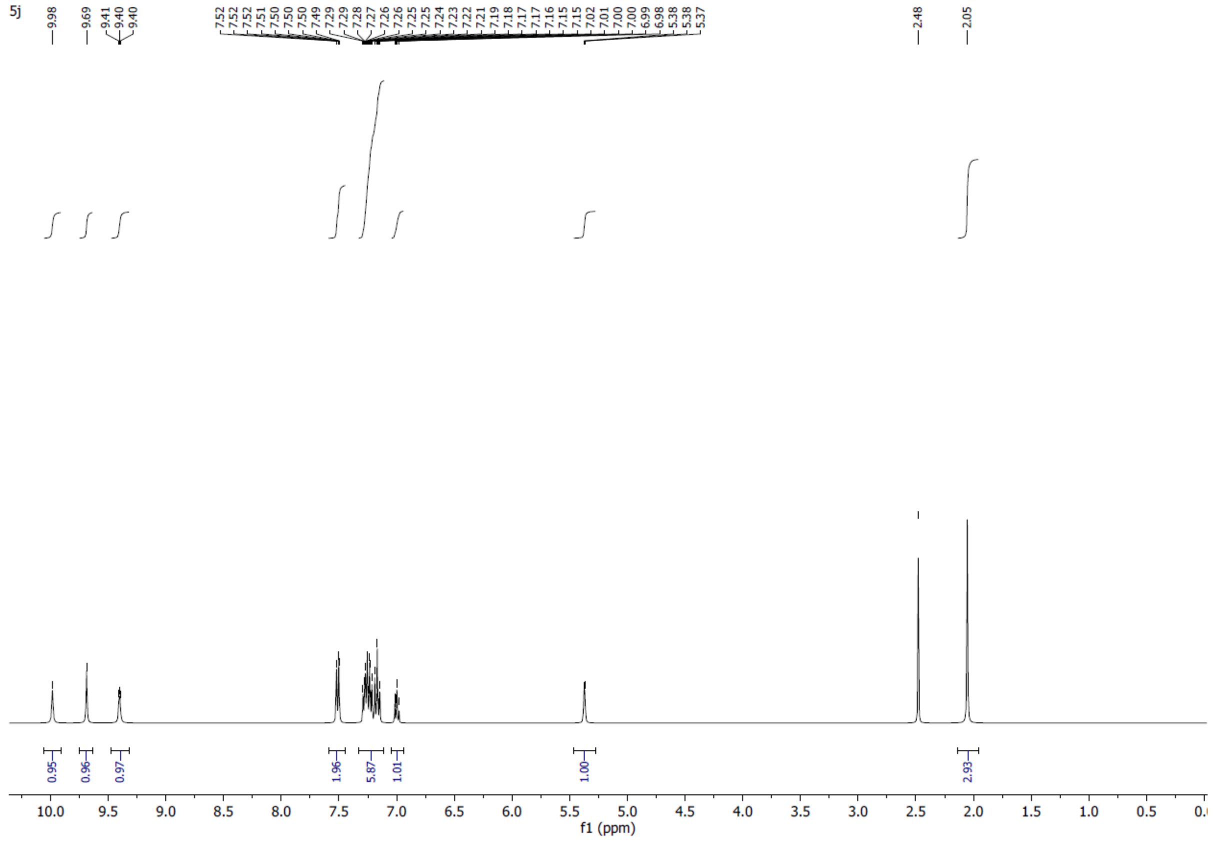

Figure S40. ^{1}H NMR (400 MHz, DMSO-*d*6) of **5j.**

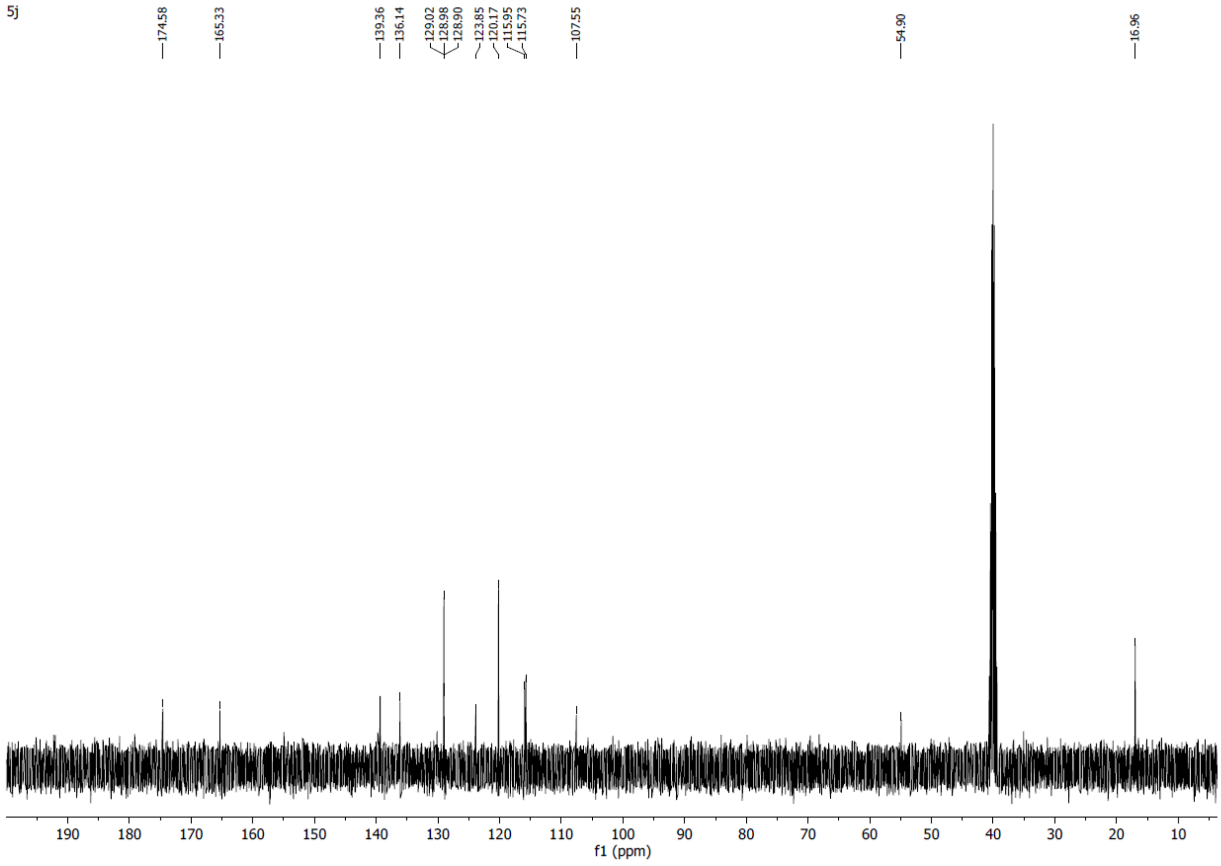

Figure S41. ^{13}C NMR (100 MHz, DMSO-*d*6) of **5j.**

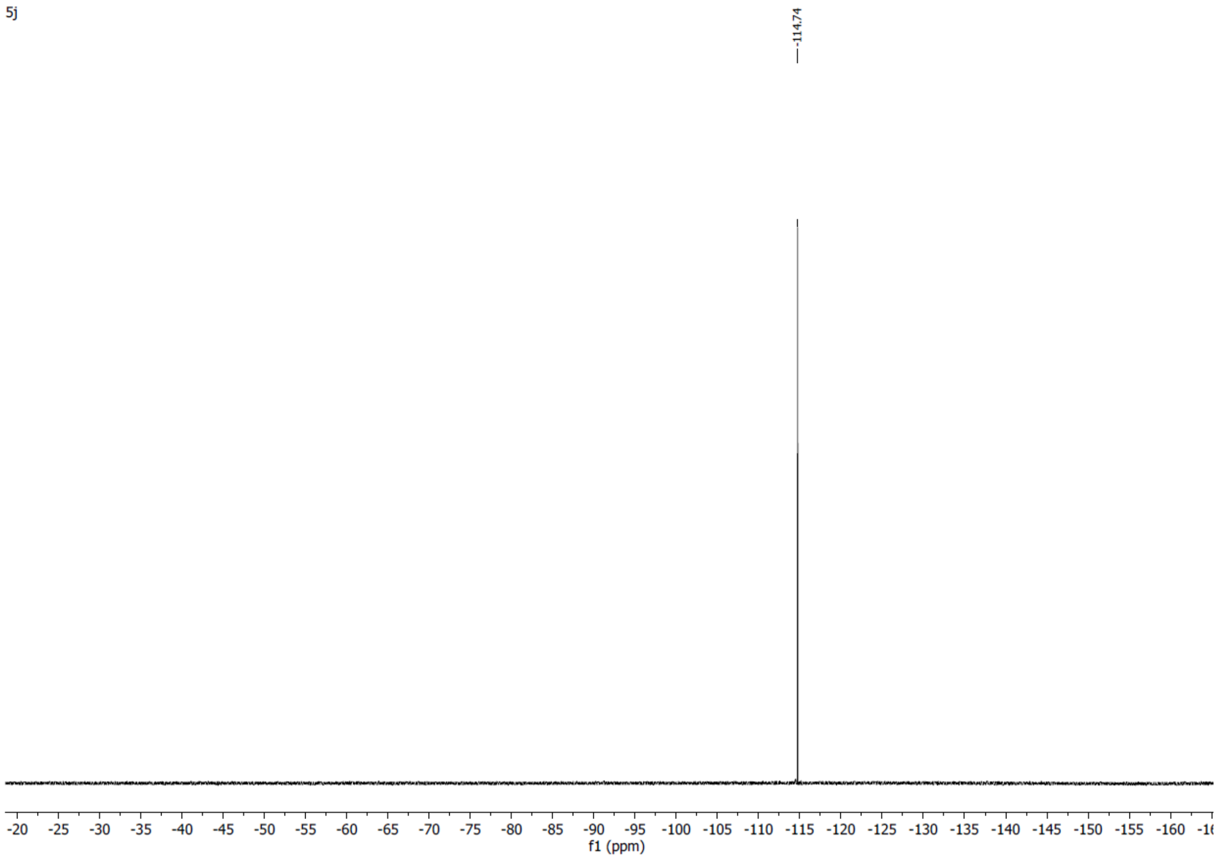

Figure S42. ^{19}F NMR (376 MHz, DMSO-*d*6) of **5j.**

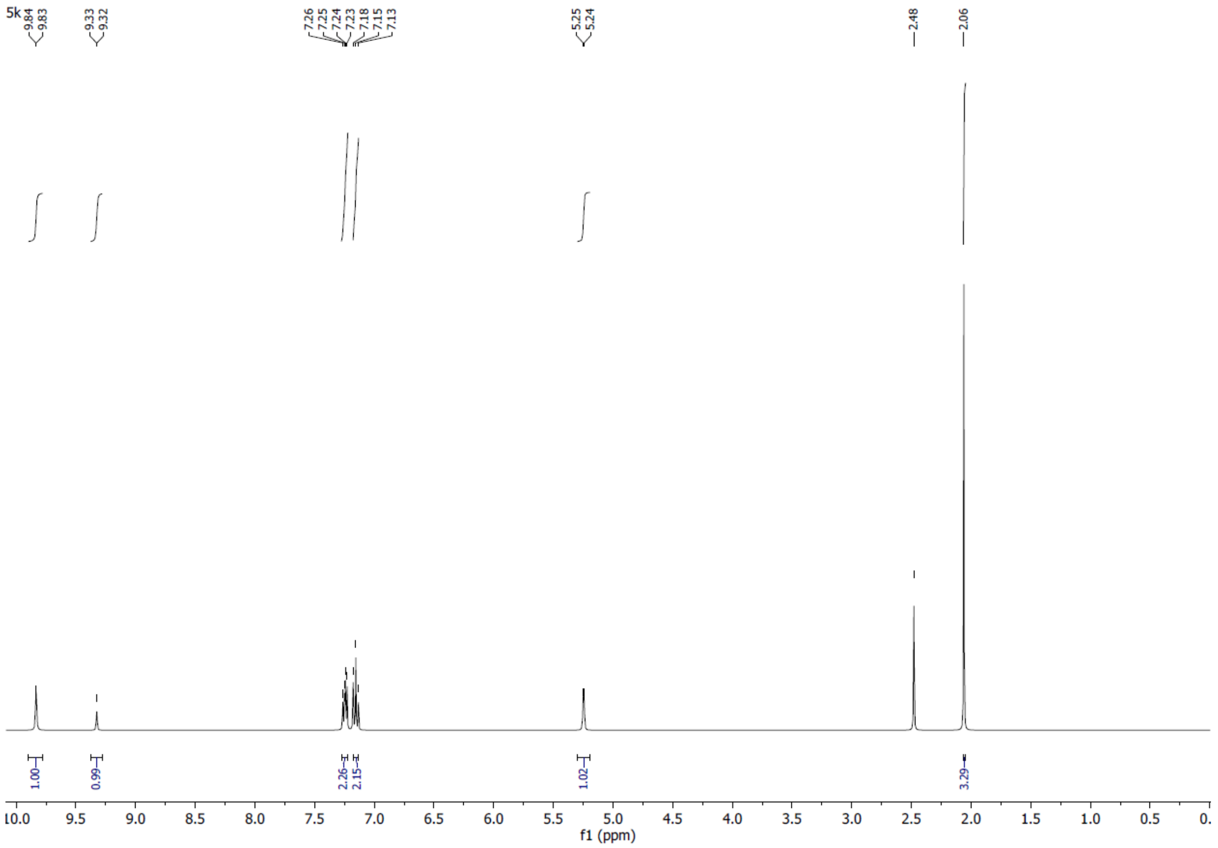

Figure S43. ^{1}H NMR (400 MHz, DMSO-*d*6) of **5k.**

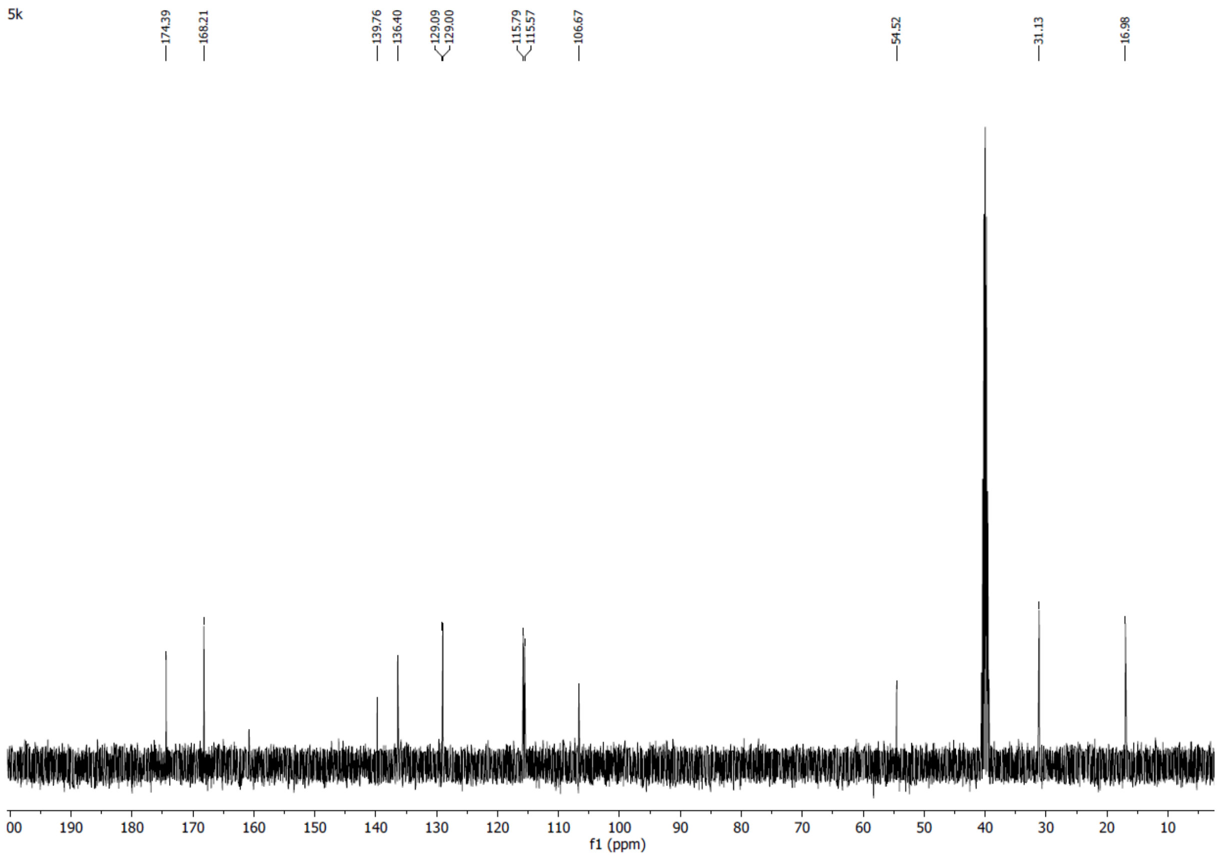

Figure S44. ^{13}C NMR (100 MHz, DMSO-*d*6) of **5k.**

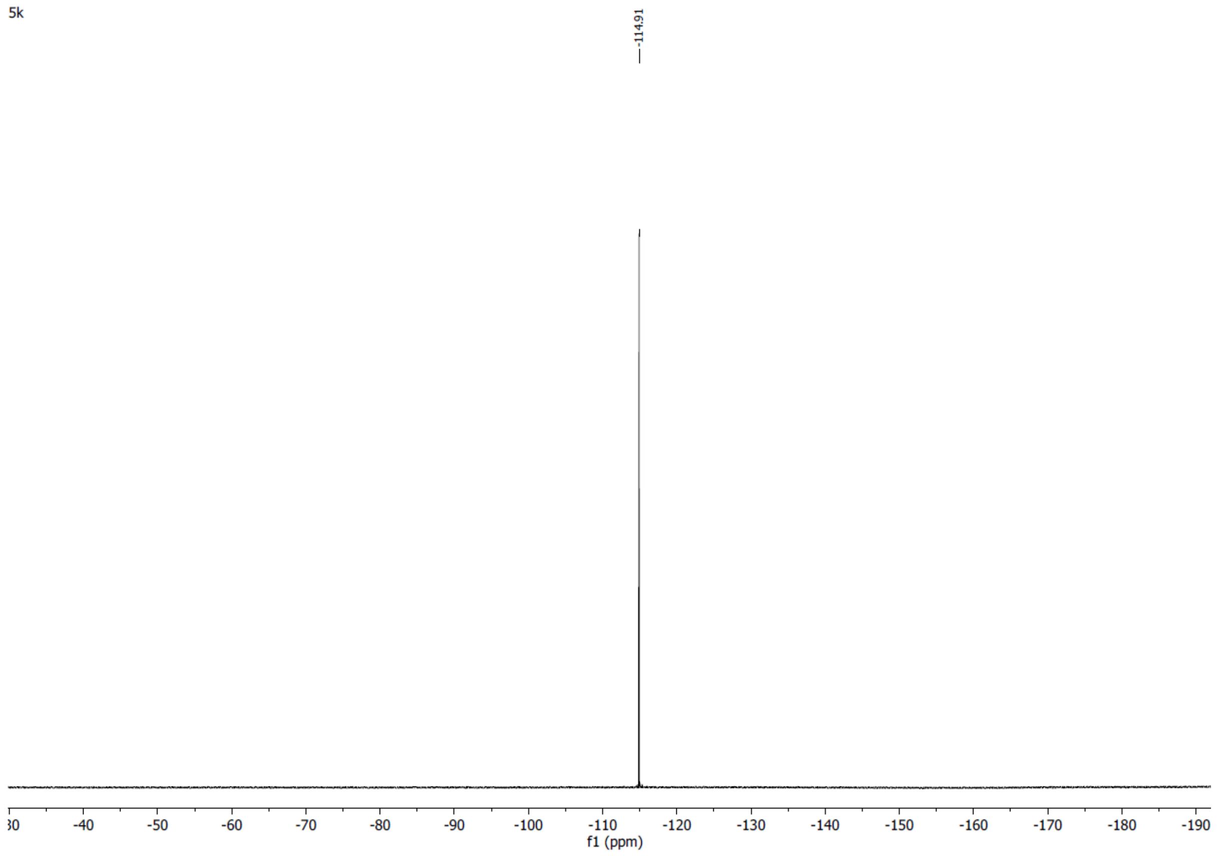

Figure S45. ^{19}F NMR (376 MHz, DMSO-*d*6) of **5k.**

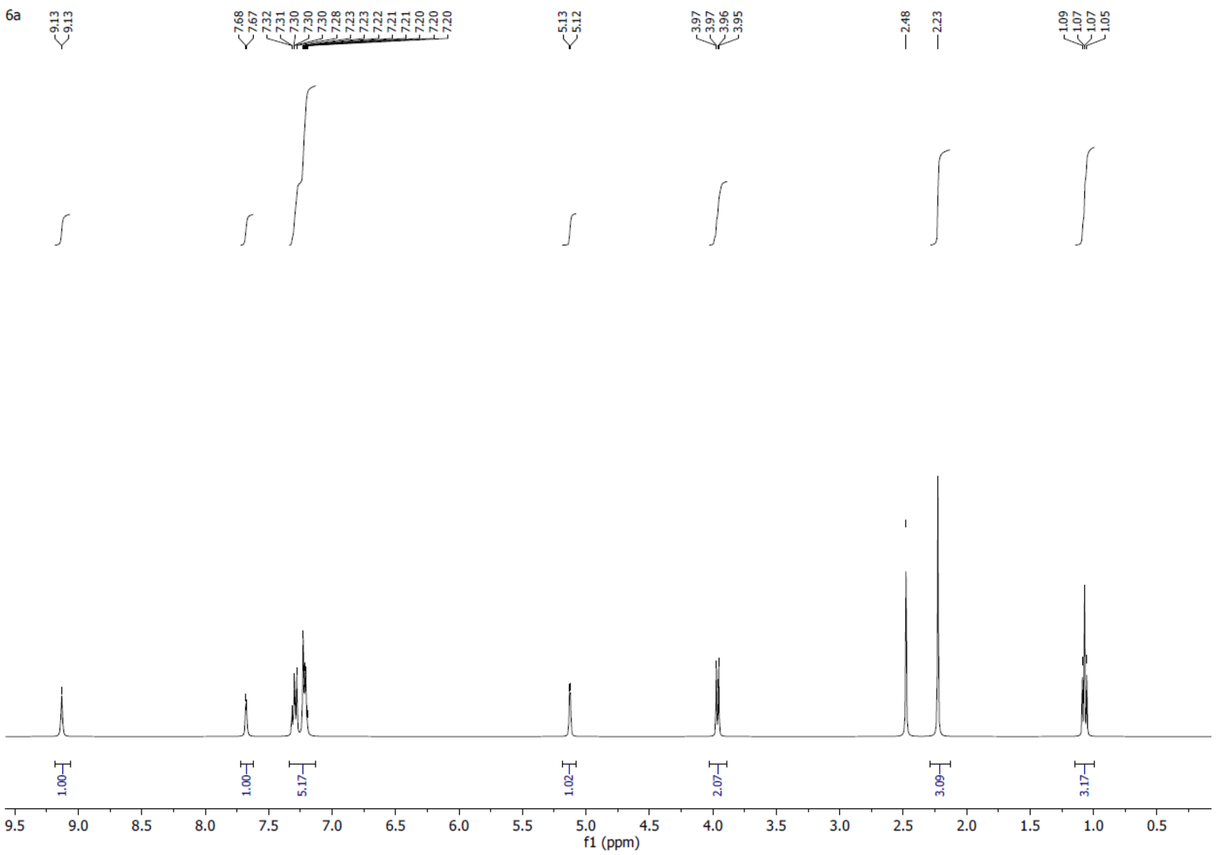

Figure S46. ^{1}H NMR (400 MHz, DMSO-*d*6) of **6a.**

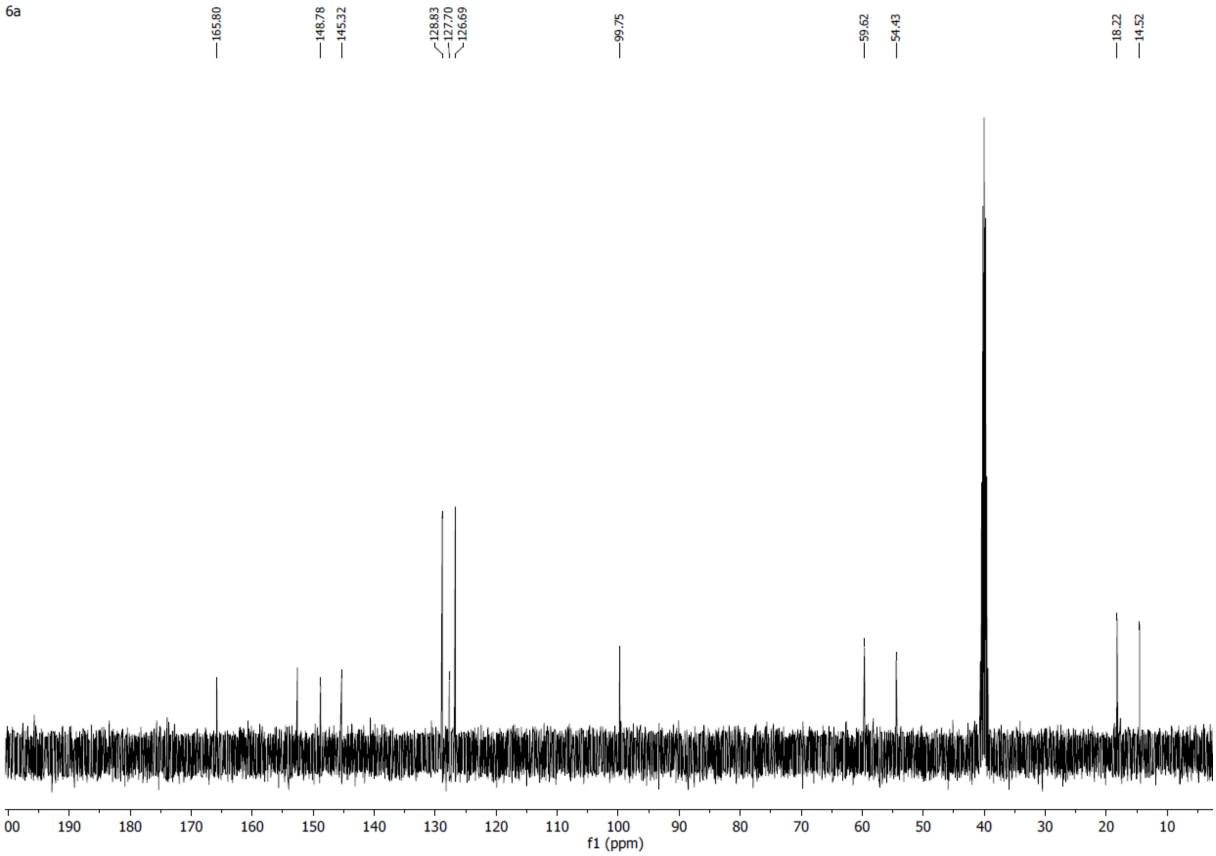

Figure S47. ^{13}C NMR (100 MHz, DMSO-*d*6) of **6a.**

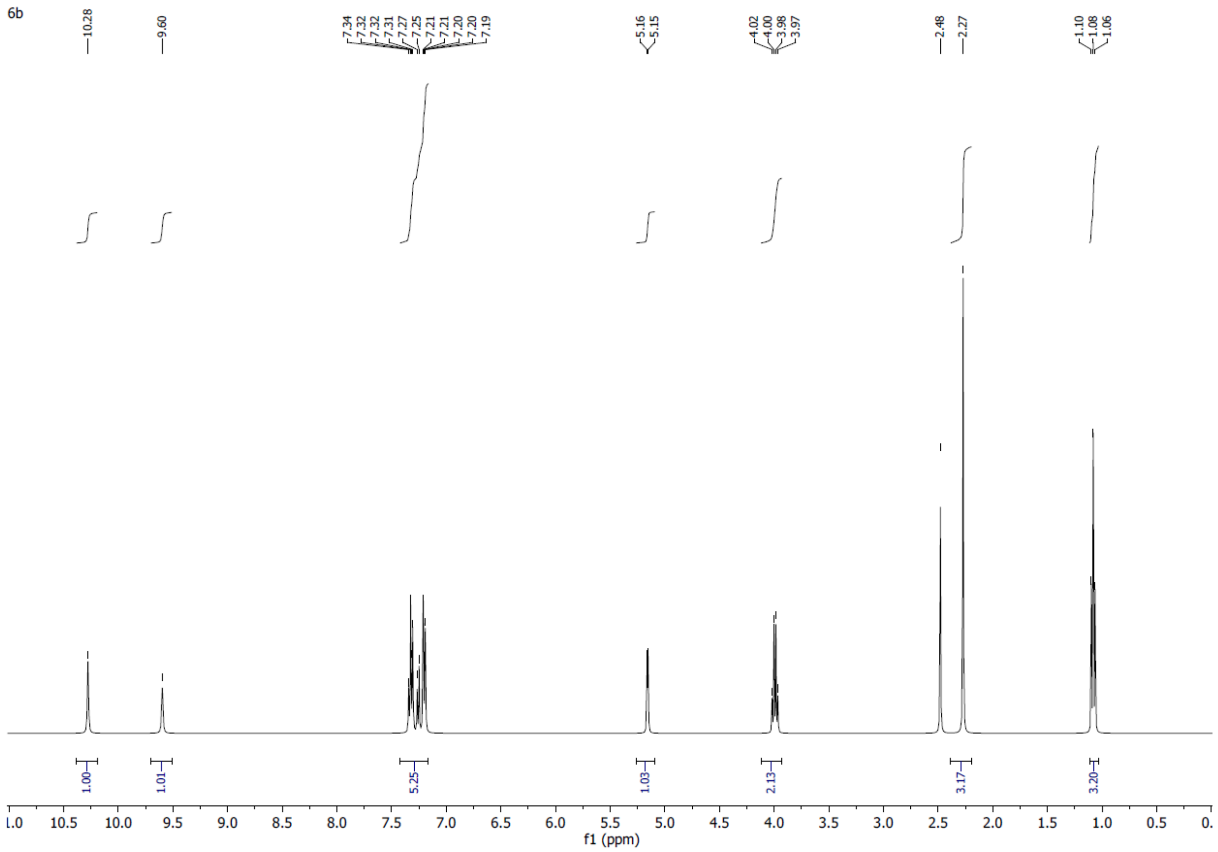

Figure S48. ^{1}H NMR (400 MHz, DMSO-*d6*) of **6b.**

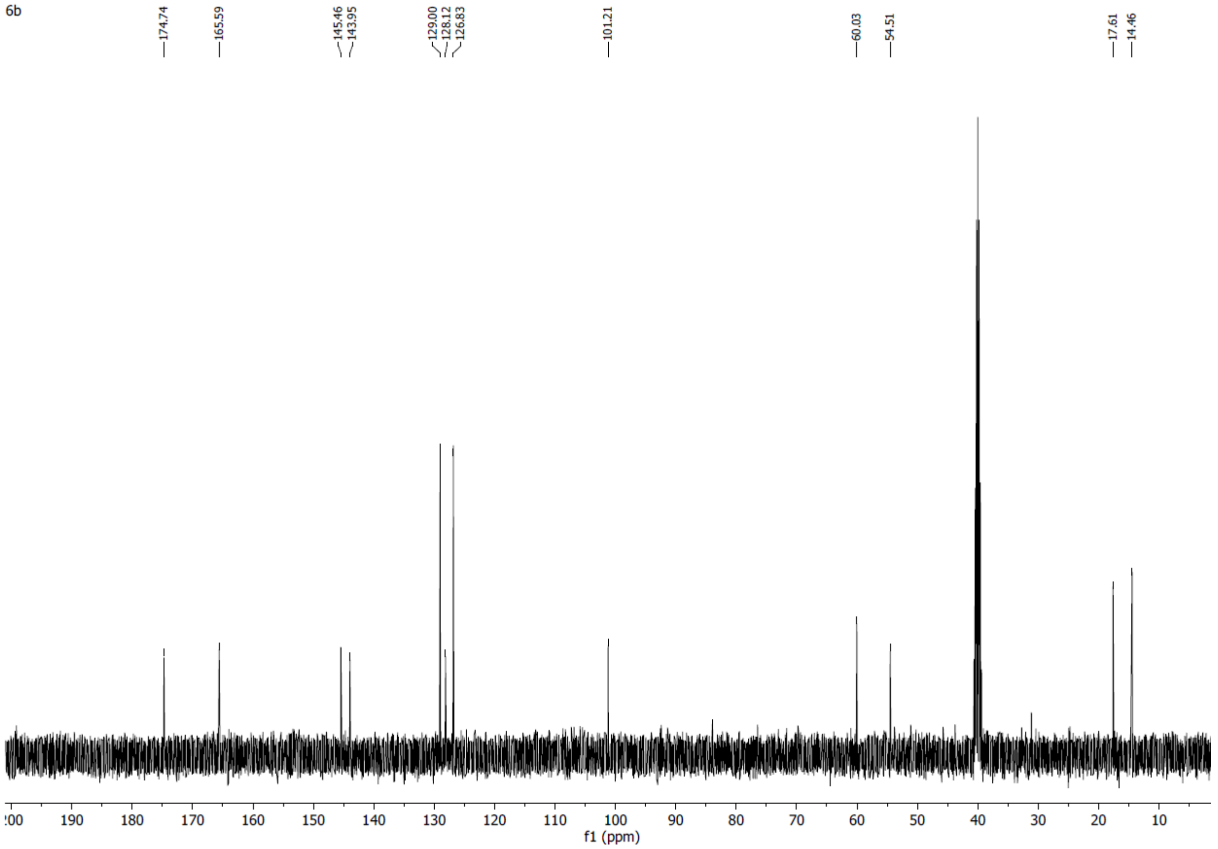

Figure S49. ^{13}C NMR (100 MHz, DMSO-*d6*) of **6b.**
